# Human Derived Immortalized Dermal Papilla Cells With a Constant Expression of Testosterone Receptor

**DOI:** 10.3389/fcell.2020.00157

**Published:** 2020-03-18

**Authors:** Tomokazu Fukuda, Kouhei Takahashi, Shin Takase, Ai Orimoto, Takahiro Eitsuka, Kiyotaka Nakagawa, Tohru Kiyono

**Affiliations:** ^1^Graduate School of Science and Engineering, Iwate University, Morioka, Japan; ^2^Soft-Path Engineering Research Center, Iwate University, Morioka, Japan; ^3^Graduate School of Agricultural Science, Tohoku University, Sendai, Japan; ^4^Division of Carcinogenesis and Cancer Prevention and Department of Cell Culture Technology, National Cancer Center Research Institute, Tokyo, Japan

**Keywords:** dihydrotestosterone, dermal papilla cells, immortalization, androgen receptor, nuclear localization

## Abstract

Androgenetic alopecia (AGA) is the most common type of hair loss, and is mainly caused by the biological effects of testosterone on dermal papilla cells (DPCs). *In vitro* culturing of DPCs might be a useful tool for the screening of target molecule of AGA. However, primary DPCs cannot continuously proliferate owing to cellular senescence and cell culture stress. In this study, we introduced mutant cyclin-dependent kinase 4 (CDK4), Cyclin D1, and telomerase reverse transcriptase (TERT) into DPCs. We confirmed protein expression of CDK4 and Cyclin D1, and enzymatic activity of TERT. Furthermore, we found the established cell line was free from cellular senescence. We also introduced the androgen receptor gene using a recombinant retrovirus, to compensate the transcriptional suppressed endogenous androgen receptor in the process of cell proliferation. Furthermore, we detected the efficient nuclear translocation of androgen receptor into the nucleus after the treatment of dihydrotestosterone, indicating the functionality of our introduced receptor. Our established cell line is a useful tool to identify the downstream signaling pathway, which activated by the testosterone.

## Introduction

Hair plays an important role in protecting the skin from mechanical damage. The hair follicle regulates hair growth through complex interactions between hormones, neuropeptides, and immune cells. The hair follicle consists of multiple cell types, including dermal papilla cells (DPCs), matrix cells, and melanocytes (Driskell et al., 2011). The process of hair growth can be classified into three stages: the growth stage (anagen), the regression stage (catagen), and the rest stage (telogen) (Milner et al., 2002). Hair growth occurs throughout the duration of the anagen phase until the hair enters the catagen phase, in which the hair follicle shrinks, and apoptosis occurs. During the telogen phase, the hair follicle remains dormant while new hair growth begins, eventually causing hair loss or “shedding” of the old hair. This hair growth and replacement process is known as the hair cycle (Davis, 1962). Hair growth and proliferation is mainly due to the hair matrix cell proliferation and differentiation into the hair shaft (comprising three layers: the medulla, cortex, and hair cuticle) and the inner root sheath (IRS, comprising three layers: the cuticle, Huxley’s layer, and Henle’s layer) (Detmar et al., 1993). The proliferation of hair matrix cells is regulated by growth stimulation from DPCs via insulin growth factor I (IGF-I) (Danilenko et al., 1996) and fibroblast growth factors 5 and 7 (FGF5 and FGF7) (Hébert et al., 1994; Guo et al., 1996).

Androgenetic alopecia (AGA) is the most common type of hair loss in men. Fifty eight percent of the 30–50-year-old men suffer from AGA (Shankar et al., 2009). AGA is characterized by hair loss from the top and front of the head. Although the molecular mechanisms of AGA are not fully understood, testosterone and its metabolic form, dihydrotestosterone (DHT), are considered to be the major causes of AGA (Kwack et al., 2008). DHT is produced from enzymatic catalysis of testosterone via the enzyme 5α-reductase (Münster et al., 2003). DHT has a stronger affinity for the androgen receptor than does testosterone, and forms a DHT-androgen receptor complex, which then moves from the cytoplasm into the nucleus. Nuclear localization of the DHT-androgen receptor complex enhances the secretion of hair growth-suppressive factors from DPCs, such as transforming growth factor β (TGFβ) or Dickkopf-related protein 1 (Dkk1) (Kwack et al., 2010). As a result, the duration of the hair growth period decreases, resulting in the loss of hair from the top and front of the head, which is characteristic of AGA.

The standard pharmaceutical product used to treat AGA is Finasteride (Gupta and Charrette, 2014). Finasteride is an inhibitor of the 5α-reductase enzyme. By inhibiting the enzymatic activity of 5α-reductase, Finasteride reduces the concentration of DHT, which prevents the progression of AGA and hair loss (Kaufman and Dawber, 1999). Although Finasteride is effective in preventing the progression of AGA, there are several major side effects. One example of the adverse effects of Finasteride is sexual dysfunction, such as erectile dysfunction or decreased libido (Coskuner et al., 2019). In addition to negative side effects, the cost of Finasteride therapy is relatively high. Consequently, the screening of new molecular target for the treatment AGA is worth investigating to reduce potential side effects and cost. Furthermore, owing to the large population of AGA patients, the discovery of new anti-AGA drugs could be very profitable.

In general, cell culture is more advantageous than traditional experimental animal models from view point of animal welfare and cost. As described previously, DPCs are the control center for regulating hair growth and are target cells for the biological effect of DHT (Kwack et al., 2008). Therefore, *in vitro* culture of DPCs would be useful to find out the molecular target and the screening of pharmaceutical products to treat AGA. DPCs can be prepared from primary cultures of human cells, but sampling and primary cell culture can produce wide variability depending on cell preparation (Topouzi et al., 2017). Furthermore, primary DPCs cannot continuously proliferate because of cellular senescence and the Hayflick limit. Owing to this limitation, the number of passages of primary DPCs could affect the results obtained.

Our research group previously reported that combined expression of R24C mutant cyclin-dependent kinase 4 (CDK4), Cyclin D1, and telomere reverse transcriptase (TERT) allowed us to efficiently immortalize human- (Shiomi et al., 2011), cattle and pig- (Donai et al., 2014), prairie vole- (Katayama et al., 2016, 2017), monkey- (Kuroda et al., 2015a), midget buffalo- (Fukuda et al., 2016), and mega bat- (Tani et al., 2019), Tsushima wildcat-derived cells (Gouko et al., 2018). Furthermore, growth acceleration with mutant CDK4 and Cyclin D1 is conserved even in sea turtles, suggesting that the underlying cell cycle mechanism was well-conserved throughout animal evolution (Fukuda et al., 2018). Cells immortalized using this method maintain the cell differentiation and chromosome patterns of the original cells (Shiomi et al., 2011). In this study, we introduced an expression cassette of R24C mutant CDK4, Cyclin D1, and TERT into human DPCs via lentivirus. Immortalized DPCs could be shared with scientists worldwide as research materials, which would contribute to experimental reproducibility. Establishment of an immortalized cell line can also reduce the necessity for primary cell culture if the original nature of the cells is preserved. Owing to the nature of DPCs, the expression of androgen receptors decreases with increasing passage number. To overcome this limitation, we also introduced an androgen receptor expression cassette through retroviral expression. This study is the first to describe the establishment of immortalized DPCs with intact chromosome condition and androgen receptor expression.

## Materials and Methods

### Cell Culture

Human follicle DPCs were obtained from PromoCell (C-12072, Heidelberg, Germany) through the local distributor, Takara Bio (Shiga, Japan). DPCs were cultured in follicle dermal papilla cell medium (cat. no, C-26500, PromoCell) supplemented with: a supplement mix containing 0.04 mL/mL fetal calf serum, 0.004 mL/mL bovine pituitary extract, 1 ng/mL basic fibroblast growth factor, and 5 μg/mL insulin; and 1% antibiotic antimycotic mixed stock solution (cat. no, 09366-44, Nacalai Tesque, Kyoto, Japan). The cell culture conditions were as follows: DPCs were seeded in a six-well plate with 2 mL of medium per well. The cells were cultured at 37°C in a humidified atmosphere with 5% CO_2_.

### Preparation of Recombinant Viruses and Genetic Introduction

To immortalize primary DPCs, we prepared recombinant lentiviruses expressing mutant CDK4, Cyclin D1, and TERT. We made a mixture of lentivirus solution expressing mutant CDK4, Cyclin D1, and TERT. The packaging of recombinant lentiviruses was carried out via transient expression of CSII-CMV-hTERT, CSII-CMV-Cyclin D1, and CSII-CMV-hCDK4R24C using packaging plasmids (pCAG-HIV-gp, pCMV-VSV-G-Rsv-Rev, kindly provided by Dr. Hiroyuki Miyoshi, Keio University, Japan) in 293T cells. As a surrogate marker to monitor the efficiency of infection, we used pCSII-CMV-EGFP, which expresses a fluorescent marker. The preparation and recombination of lentiviruses was previously described in our past study (Fukuda et al., 2017). We named the cells transfected with R24C mutant CDK4, Cyclin D1, and TERT as K4DT cells, in reference to the modified genes (Katayama et al., 2019). We similarly named the recombinant cells with R24C mutant CDK4 and Cyclin D1 as K4D cells. The recombinant cells were automatically selected by the growth advantage, we carried out the cell analysis based on the pooled cell population.

To express the androgen receptor in our established K4DT cells, we chemically synthesized an expression cassette of the androgen receptor, including hemagglutinin (HA) at the amino terminal end. HA was used as a tag for convenient detection of the introduced androgen receptor protein. We inserted the cDNA fragment encoding the androgen receptor with an HA tag into the multiple cloning site of QCXIN, a retroviral vector (Takara Bio). As a surrogate marker to monitor the efficiency of infection, we used QCXIN-EGFP, which expresses EGFP. The packaging of retrovirus was carried out in 293T cell under the transient expression of QCXIN-AR or QCXIN-EGFP, and packaging plasmids, pCL-gag pol, and pCMV-VSV-G-RSV-Rev (Dr. Hiroyuki Miyoshi, RIKEN BioResorce Center, Tsukuba, Japan). The detailed packaging procedure was described in our previous report (Fukuda et al., 2018). We named immortalized DPCs with androgen receptor expression K4DT-AR, and immortalized DPCs with control EGFP expression K4DT-EGFP.

### Western Blotting

We carried out western blotting to detect the expression of proteins encoded by the introduced genes, i.e., CDK4, Cyclin D1, and the androgen receptor. The cells were lysed in a solution containing 50 mM Tris-HCl (pH 7.4), 0.15 M NaCl, 1% Triton X-100, 2.5 mg/mL sodium deoxycholate, and a protease inhibitor cocktail. A detailed protocol for western blotting is described in our previous study (Fukuda et al., 2017).

A rabbit anti-human Cyclin D1 antibody (1:5000, code no. 553, Medical & Biological Laboratories Co., LTD., Nagoya, Japan), a mouse anti-human CDK4 antibody (1:200, cat. no. sc-56277, Santa Cruz Biotechnology, Dallas, TX, United States), and a mouse anti-α-tubulin antibody (1:1000, cat. no. sc-32293, Santa Cruz Biotechnology) were used as primary antibodies. An anti-HA high affinity antibody (cat. no. 11867423001, clone 3F10, 100 μg/mL solution, Sigma Aldrich, St. Louis, MO, United States) was used for the western blotting at a finale concentration of 100 ng/mL. A sheep anti-mouse IgG-linked horseradish peroxidase (HRP) (1:2000, code no. NA931V, GE Healthcare, Buckinghamshire, United Kingdom), a donkey anti-rabbit IgG-linked HRP (1:2000, code no. NA934V, GE Healthcare), and an anti-rat IgG-linked HRP (1:2000, code no. 31470, Thermo Fisher Scientific, Waltham, MA, United States) were used as secondary antibodies. Signals from the target proteins were detected using Signal Enhancer HIKARI for Western Blotting and ELISA (code no. 02270-81, Nacalai Tesque), and the images were detected using an ImageQuant LAS-4000 Mini system (GE Healthcare).

### Immunofluorescence Staining of HA Tag

To examine the localization of the androgen receptor in our established cells, we carried out immunofluorescence staining. Cells were cultured on a chamber slide (cat. No. 177473, Thermo Fisher Scientific). Forty-eight hours after seeding, we fixed the cells with 4% paraformaldehyde in phosphate-buffered saline (PBS) (cat. no, 09154-85, Nacalai Tesque). Permeabilization was performed by incubating cells in PBS with 0.5% Triton X-100 for 15 min (cat. no, 35501-15, Nacalai Tesque). After washing with PBS with 0.1% Tween 20 for 5 min (lot. no, M8M7587, Nacalai Tesque), we incubated the cells in a 1% bovine serum albumin blocking solution (BSA, cat. no, 01863-06, Nacalai Tesque) on a rotary shaker (NR-2, TAITEC, Saitama, Japan) for 45 min. After blocking, cells were exposed to the primary antibody overnight. The anti-HA high affinity antibody (cat. no. 11867423001, clone 3F10, 100 μg/mL solution, Sigma-Aldrich, St. Louis, MO, United States) was diluted at 1:20 in PBS with 1% BSA. After primary antibody incubation, cells were incubated with the second antibody, which acts as a fluorescent marker. A goat anti-rat IgG conjugated with Alexa Fluor 568 (cat. no. A11077, Thermo Fisher Scientific) was used as a secondary antibody to detect the HA-tag. DAPI (4′,6-diamidino-2-phenylindole) was used as a counterstaining reagent.

### Senescence-Associated β-Galactosidase (SA-β-Gal) Staining

At passage number 13, we carried out staining of senescence-associated β-galactosidase (SA-β-Gal) in K4DT cells, K4D cells, and primary DPCs. We conducted staining procedures based on the protocol described by Debacq-Chainiaux et al. (2009).

### Genomic PCR

To extract genomic DNA from cells, we used the NucleoSpin Tissue Kit (cat. no. 740952, Takara Bio). PCR was performed with 100 ng of template DNA, 1X KOD-FX neo PCR buffer (KFX- 201; Toyobo, Osaka, Japan), 0.4 mM dNTPs (KFX-201, Toyobo), 0.5 U KOD-FX neo (KFX-201, Toyobo), and 0.3 mM of each primer, in accordance with the manufacturer’s protocol. PCR was carried out under the following conditions for 40 cycles: pre-denaturation at 94°C for 2 min, denaturation at 98°C for 10 s, and extension at 68°C for 1 min (two-step PCR). Tuberous sclerosis type II (TSC2) was used as an internal control since TSC2 does not have any pseudogenes in its genome. We analyzed the PCR products by electrophoresis in 0.8% agarose/Tris-acetate–EDTA (ethylenediaminetetraacetic acid) gels and stained with ethidium bromide (14603-64, Nacalai Tesque). We used the same forward primer for the detection of the Cyclin D1, CDK4, and TERT expression cassettes: 5′-GGCACCAAAATCAA CGGGACTTT-3′. The reverse primer for the detection of Cyclin D1 was 5′-TTCCTCGCAGACCTCCAGCA-3′. The reverse primer for the detection of CDK4 was TF808, 5′-ACGAACTGTGCTGATGGGAAGGC-3′. The reverse primer for the detection of TERT was TF809, 5′-AGCTCCTTCAGGCAGGACACCT-3′. To detect TSC2, the forward primer 5′-AAACCGAGCCCCATTTGACC-3′ and the reverse primer 5′-TGGTCGTAGCGGAATCGAGGAT-3′ were used.

### Cell Cycle Analysis

We performed cell cycle analysis of primary, K4D, and K4DT cells at passage number 5 using a Muse Cell Cycle Assay Kit (cat. no. MCH100106, Merck Millipore Corporation, Billerica, MA, United States) and a Muse Cell Analyzer (cat. no. 0500-3115, Merck Millipore Corporation). The fixation and analysis procedures are described in the protocol provided by the manufacturer. The statistical significance of each cell cycle stage was evaluated using the non-parametric multiple comparison method (Steel test). A *p*-value < 0.05 was considered statistically significant (*n* = 6).

### Alkaline Phosphatase Staining

Cells were fixed with 4% paraformaldehyde in phosphate-buffered saline (PBS) (09154-85; Nacalai Tesque) for 3 min and then incubated with an alkaline phosphatase staining solution at 37°C for 10–15 min. The staining solution consisted of 0.6 mg/mL Fast Red TR Salt [hemi(zinc chloride) salt, F8764; Sigma-Aldrich, St. Louis, MO, United States], 0.1 mg/mL naphthol phosphate (23821-24; Nacalai Tesque), 0.7 mM N,N-dimethylformamide (13016-65; Nacalai Tesque), 0.2 mM MgCl_2_ (20908-65; Nacalai Tesque), and 0.1 mM Tris-HCl (pH 8.5). The staining reaction stopped after ∼15 min due to fixation with the 4% paraformaldehyde solution. More details are described in our previous reports (Donai et al., 2013).

### Population Doubling Assay

Primary, K4D, and K4DT cells were seeded at densities of 5 × 10^4^ cells/well in 35 mm diameter dishes (Thermo Fisher Scientific). Experiments were carried out in triplicate. When the cells in one of the wells reached confluence, all the cells were passaged, and then the total number of cells in each well was counted using an automated cell counter (Countess, Thermo Fisher Scientific). Population doubling (PD) was a measure of cell growth rate. PD was calculated with the following formula: PD = log_2_(a/b), where a is the total number of cells in the current passage and b is the number of seeded cells (Qin et al., 2012). The averages and standard deviations (SD) were calculated from triplicate samples.

### Karyotype Analysis

Karyotype analysis was carried out in K4DT cells. K4DT cells were treated with 0.02 mg/mL colcemid (Sigma-Aldrich) overnight. After trypsinization, cells were exposed to a hypotonic solution and fixed in Carnoy’s fluid. After fixing, cells were stained with Giemsa solution (Fukuda et al., 2012b). The chromosome number was determined from 50 mitotic cells, and the detailed chromosomal condition was evaluated using G-banding in 20 mitotic cells.

### Detection of Endogenous Androgen Receptor Gene With Real Time PCR

To address the expression level of androgen receptor in cells, we carried out the detection of mRNA level of endogenous androgen receptor gene with real time PCR analysis. Total RNAs were extracted from cells at 80% confluent condition in 35 mm dishes. Total RNAs were obtained from wild type DPCs at passage 8, DPCs with K4DT expression at passage 12, human embryonic fibroblasts HE16 (RIKEN BioResource Research Center, Tsukuba, Japan) with NucleoSpin RNA Plus (Takara Bio). As the positive control for detection, we purchased human prostate total RNA (Takara Bio, Z6550N). Five microgram of total RNAs were used for the reverse transcription reaction with PrimeScript RT reagent Kit with gDNA Eraser, Perfect Real Time (Takara Bio, RR047A) with random hexamer primer in 25 μL of volume. One microliter of cDNAs were applied into the real time PCR reaction with specific primers for androgen receptors (TF1007; 5′-CCAGCAGAAATGATTGCAC-3′, and TF1008; 5′-ATTACCAAGTTTCTTCAGCTTC-3′) with Thumderbird Sybr qPCR mix (Toyobo) and Thermal Cycler Dice^®^ Real Time System II (Takara Bio, TP960). The expression level of androgen receptor was adjusted with the level of house keeping gene, glyceraldehyde-3-phosphate dehydrogenase (GAPDH) detected with specific primers (TF999; 5′- GAGGTGCACCACCAACTGCTTAGC-3′ and TF1000; 5′-TCGGCATGGACTGTGGTCATGAG-3′). The average expression level and standard error of each samples were calculated from six replicates for each group.

### F-Actin Distribution Detected by Fluorescence Phalloidin

We carried out the cell culture of wild type, K4DT, and AR expressing K4DT DPCs in 35 mm glass base dish (code 3970-035, Iwaki, Shizuoka, Japan) after the collagen treatment. Around 1.0 × 10^5^ cells of wild type, K4DT, AR expressing K4DT DPCs cells were seeded into each glass base dish. After 2 days later, the cells were washed by 1X PBS, then fixed with 4% paraformaldehyde solution. After the permeabilized treatment with Triton X-100, we stained the cells with X40 diluted Rohodamine X conjugated Phalloidin solution (code 165-2164, FUJIFILM Wako Pure Chemical, Osaka, Japan, powder was dissolved into 1.5 mL of dimethylformamide, final concentration is 6.6 μmol/L). We detected the intensity of fluorescence images with imageJ software. The intensity was detected from the cytoplasm of 15 randomly selected cells.

### Immunostaining of α-Smooth Muscle Actin (SMA)

For the characterization of DPCs, α-smooth muscle actin (SMA) is one of the marker genes. To detect the immune-staining, we used primary antibody against SMA (sc-32251, Santa Cruz Biotechnology, Dallas, TX, United States). The seeding condition is identical with that of F-actin in the previous section. Cells were washed by 1X PBS, then fixed with 4% paraformaldehyde solution. After the permeabilized treatment with Triton X-100, we did the blocking with 1% bovine serum albumin in PBS for 10 min. Next, X100 diluted primary antibody was exposed to the cell overnight at 4°C. After the wash with 1X PBS wash, the secondary antibody, Goat anti-Mouse IgG (H + L) with Alexa 488 (Thermo Fisher Scientific) was exposed to the cells with X 200 dilution for 1 h. DAPI (4′,6-diamidino-2-phenylindole) was used as a counterstaining reagent. We detected the intensity of fluorescence images with imageJ software. The intensity was detected from the cytoplasm of 15 randomly selected cells.

### Protocol for DHT Treatment and Real Time PCR Detection of Dkk1 and TGFβ1

For the dihydrotestosterone treatment, cell culture medium for cell growth has replaced into DMEM/F12 medium (code 048-29785, FUJIFILM Wako Pure Chemical) without serum, with antibiotics for 48 h. The dihydrotestosterone (DHT) (Stanolone, code A0462, Tokyo Chemical Industry, Tokyo, Japan) was dissolved in Dimethyl sulfoxide at 100 μM concentration. 1/1000 volume of DHT solution was added to DMEM/F12 without serum and exposed to the cells for 48 h. We obtained total RNA with NucleoSpin RNA Plus (Takara Bio). Five microgram of total RNAs were used for the reverse transcription reaction with PrimeScript RT reagent Kit with gDNA Eraser, Perfect Real Time (Takara Bio, RR047A) with random hexamer primer in 25 μL of volume. One microliter of cDNAs were applied into the real time PCR reaction with specific primers for Dkk1 (TF1041; 5′-GCGGGAATAAGTACCAGAC-3′, TF1042; 5′-CGCAGTACTCATCAGTGCC-3′, Taqman probe; 5′FAM-AACTACCAGCCGTACCCGTGC-3′BHQ) with Thumderbird Probe qPCR mix (Toyobo) and Thermal Cycler Dice Real Time System II (Takara Bio, TP960). The expression level of Dkk1 was adjusted with the level of housekeeping gene, glyceraldehyde-3-phosphate dehydrogenase (GAPDH) detected with specific primers. The average expression level and standard error of each samples were calculated from six replicates for each group. For the detection of expression level of TGFβ1, specific primers were used for the detection (TF1075; 5′-CCGAGCCCTGGACACCAAC-3′ and TF1076; 5′-CACTTCCAGCCGAGGTCCTT-3′) with Thumderbird Syber qPCR mix (Toyobo).

### The Detection of Nuclear Translocation of AR After the DHT Treatment

Presence and absence of DHT, we stained AR expressing K4DT DPCs with HA antibody. We obtained 16 cell images from each group. Based on the staining feature, we counted the number of cells, which is both positive for nuclear and cytoplasm. Furthermore, the number of cells, which is positive only within the nuclear was also counted in 16 images. Based on the number of positive cells, the ratio of cells, which both positive for nuclear and cytoplasm, the ratio of cells, which only positive for nuclear, were calculated. MDV3100 (code 11596, Cayman Chemical, Ann Arbor, MI, United States) was dissolved in DMSO at 10 mM solution. The cell was exposed at 10 μM sokution under the presence of 100 nM DHT.

### Statistical Analysis

To evaluate statistical differences, we used the non-parametric method, Steel’s test or Mann–Whitney *U*-test. *P*-values that were less than 0.05 were considered statistically significant.

## Results

### Transduction With CDK4, Cyclin D1, and TERT Resulted in Immortalized DPCs

**FIGURE 1:**
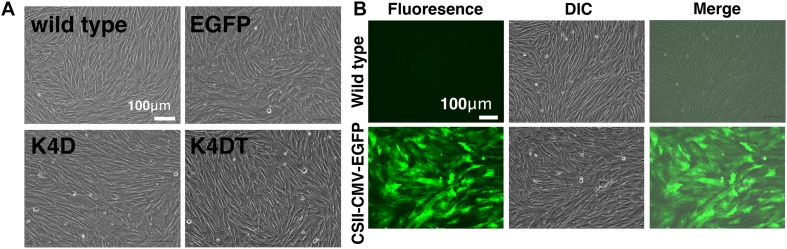
The morphologies of wild type, EGFP-expressing, mutant CDK4 and Cyclin D (K4D cell)-expressing, and mutant CDK4, Cyclin D, and telomere reverse transcriptase (TERT)-expressing cells (K4DT cells). **(A)** Cell morphology of wild type (upper left), EGFP (upper right), CDK4 and Cyclin D (lower left), and mutant CDK4, Cyclin D, and TERT-expressing cells. **(B)** Detection of EGFP fluorescence after infection of DPCs with EGFP-expressing lentiviruses. Fluorescent, difference in contrast (DIC), and merge images are shown.

We transduced EGFP or human CDK4, Cyclin D1, and TERT into primary DPCs using lentiviruses. We named the cells transfected with R24C mutant CDK4, Cyclin D1, and TERT as K4DT cells, in reference to the modified genes (Katayama et al., 2019). We similarly named the recombinant cells with R24C mutant CDK4 and Cyclin D1 as K4D cells. As shown in Figure 1A, we did not observe any differences among the cell morphologies of primary, K4D, K4DT, and EGFP cells, indicating that transduction of human CDK4, Cyclin D1, and TERT via lentiviruses did not cause any toxicity in these cells. We monitored the gene delivery efficiency of the lentiviruses in DPCs using the recombinant lentivirus CSII-CMV-EGFP as a surrogate marker. As shown in Figure 1B, cells transduced with EGFP-expressing lentiviruses showed a high level of green fluorescence, whereas wild type (uninfected control) DPCs did not. From these results, we estimated that transduction of these genes into DPCs by lentiviruses has an efficiency of 70–80%.

### Detection of the Genomic Expression Cassettes With PCR, and Detection of Protein Expression

**FIGURE 2:**
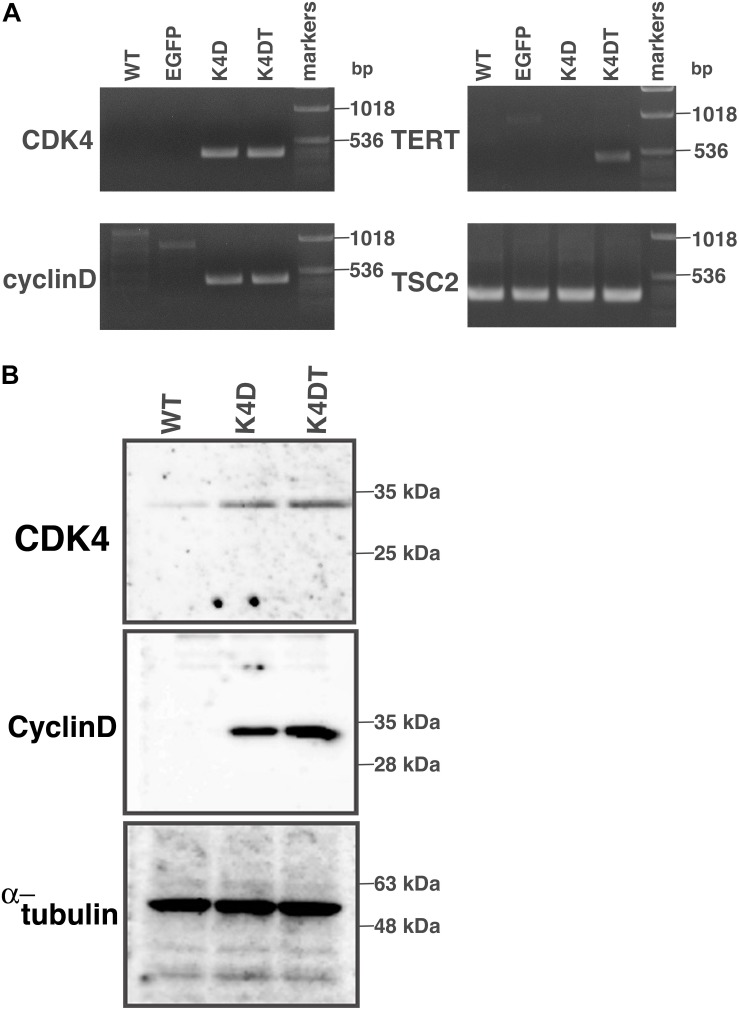
Detection of integration of the expression cassette into DPC genomes with PCR, and detection of protein expression of the introduced genes. **(A)** PCR detection of the expression cassettes of CDK4, Cyclin D, and TERT in the genomic DNA of DPCs. PCR products from Tuberous sclerosis type 2 (TSC2) were used as a control. **(B)** Western blot analysis of wild type, K4D, and K4DT cells. The results obtained from CDK4, Cyclin D, and α-tubulin antibodies are shown.

First, we carried out polymerase chain reaction (PCR) to detect integration of the genomic expression cassettes – CDK4, Cyclin D1, and TERT, introduced by lentiviral infection – into the genomic DNA of primary DPCs. We analyzed four cell populations: wild type, EGFP, K4D, and K4DT cells. The results of the PCR are shown in Figure 2A and Supplementary Figure S2. As expected, although the primary and EGFP cells (Lanes 1 and 2) did not show any product, K4D and K4DT cells (Lanes 3 and 4) showed a strong band. We used TSC2 as a control. The results of control PCR amplification showed that all genomic DNA produced sufficient amplification products, indicating that the recovery of genomic DNA and the amplification reaction worked properly.

Next, we carried out western blotting to detect the expression of CDK4 and Cyclin D1 proteins with specific antibodies (Figure 2B and Supplementary Figure S3). K4D and K4DT cells showed specific bands at the expected molecular weight (Lanes 2 and 3), whereas primary cells showed a weak signal, which could be attributed to endogenous human-derived CDK4 protein (Lane 1). We used α-tubulin as a positive control. Taken together, these data show that exogenous genes were inserted into the genomic DNA of DPCs by lentiviral transduction, and the expression cassettes produced functional proteins.

### K4DT Cells Retain the Characteristics of Primary Cells

**FIGURE 3:**
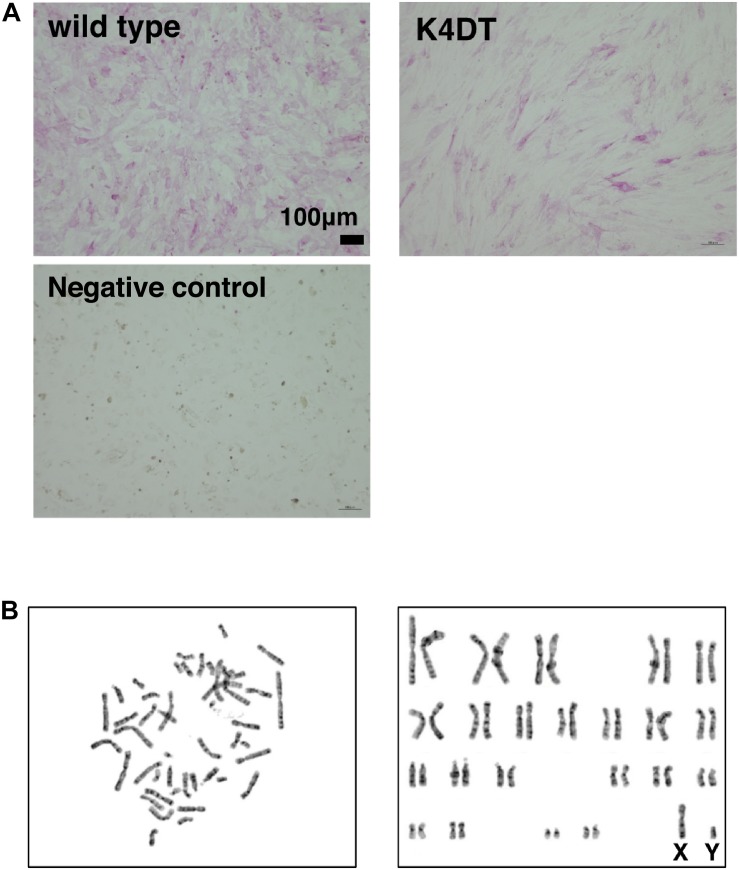
Detection of the alkaline phosphatase activity and the chromosome conditions of established DPCs. **(A)** Detection of alkaline phosphatase activity using Fast Red in wild type (upper left), K4DT (upper right), and rat-derived fibroblasts (lower right, negative control). **(B)** Chromosome analysis of K4DT cells. Left panel, a representative mitotic chromosome pattern from a K4DT cell. Right panel, an aligned G-banding chromosome pattern obtained from a K4DT cell. Sexual chromosomes are labeled as X and Y.

To elucidate whether established K4DT cells retained the characteristics of primary cells, we assayed the activity of alkaline phosphatase (AP), one of the biological markers of dermal papilla cell, at around passage 5. As shown in Figure 3A, primary cells showed positive staining for AP (Figure 3A, upper left). In addition to primary cells, we also found that K4DT cells stained positive for AP activity (Figure 3A, right panel). We confirmed the negative staining of AP in rat-derived fibroblasts, supporting the specificity of the AP staining (Figure 3A, lower left). These results showed that K4DT cells retained characteristics of the original primary cells.

### Karyotype Analysis

As shown in Figure 3B, we analyzed the chromosomal condition of K4DT cells at around passage 13 using the G-band method. We evaluated the number of chromosomes in 50 dividing K4DT cells. The karyotyping results revealed that K4DT cells were complete, normal 46XY diploids (100%, Table 1). In G-banding analysis, 19 cells showed a complete normal pattern of human chromosomes (95%), but one sample exhibited a chromosomal abnormality in chromosome 1 at the P36 position. Thus, we concluded that the expression of CDK4, Cyclin D1, and TERT allows for cellular immortalization of DPC cells with an intact chromosomal pattern.

**TABLE 1:** Karyotype analysis of our established immortalized human follicle dermal papilla cells.

Cell line	Total cell counts	Chromosome Number		
		
		45	46	47
K4DT	50	0	50	0

### K4DT Cells Were Immortalized and Overcame Cellular Senescence

**FIGURE 4:**
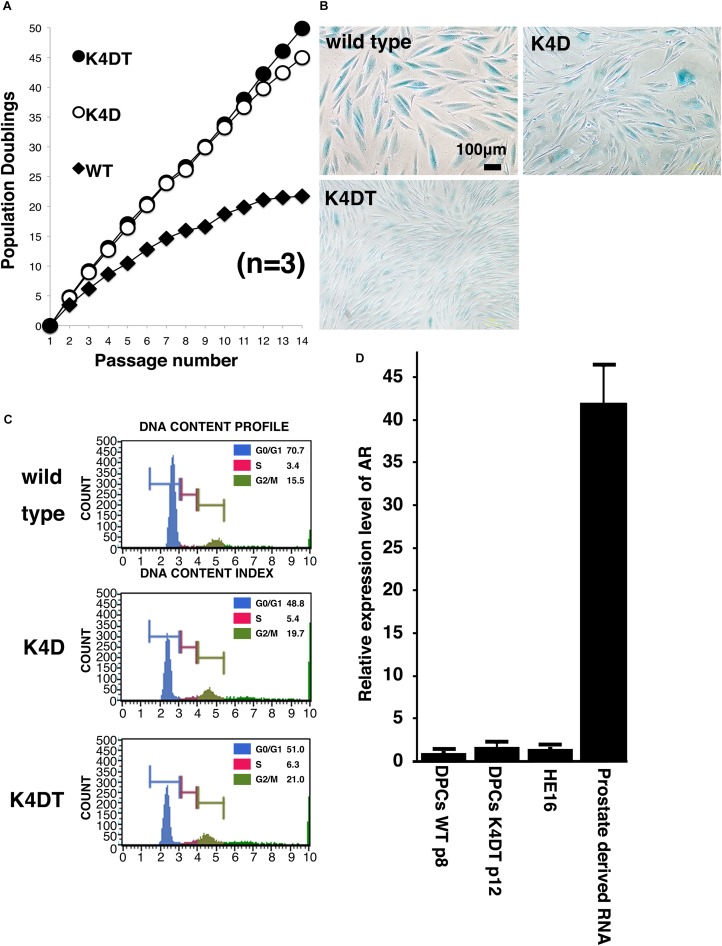
Results of sequential passaging, detection of cellular senescence, and expression levels of the androgen receptor. **(A)** Sequential passaging and cumulative population doubling of wild type, K4D, and K4DT cells. Averages and standard deviations were obtained from triplicate samples. **(B)** Detection of cellular senescence using SA-β-gal staining in wild type, K4D, and K4DT cells. The bar indicates 100 μm. **(C)** Cell cycle histogram of representative results obtained from wild type, K4D, and K4DT cells. **(D)** Detection of the androgen receptor gene in wild type DPCs at passage 8, immortalized K4DT DPCs at passage 12, HE12 human fibroblasts, and adult human prostate-derived RNA. Wild type DPC expression levels were set to 1.0, and the relative amount of AR was detected using real-time PCR. Six samples were analyzed for each group. The averages and standard deviations are shown.

To evaluate the cell proliferation rate and measure the population doubling (PD) of the established cell lines, we carried out sequential passaging. As shown in Figure 4A, although primary cells showed a gradually decrease in cell proliferation ability, K4D and K4DT cells continued to proliferate without a decrease in cell growth. However, K4D cells tended to display slower rates of proliferation at around passage 12, which could be explained by cellular senescence. As shown in Figure 4B, we detected cellular senescence in each cell line at passage 14, after serial passage, using SA-β-Gal staining. While almost all the primary cells showed intense positive staining (Figure 4B, upper left), staining signals were much weaker in K4DT cells (Figure 4B, lower left). In K4D cells, although some cells showed positive staining, the incidence of positive cells was much lower than that in primary cells (Figure 4B, upper right). The cell size of primary cells at passage 14 became much larger than that of K4DT or K4D cells, indicating that K4DT cells do not exhibit an increase in cytoplasm, which is one of the characteristics of aged cells.

### Cell Cycle Analysis

To compare the cell cycle distribution of cells, we performed cell cycle analysis using a Muse Cell Cycle Assay Kit (Merck Millipore Corporation) and a Muse Cell Analyzer (Merck Millipore Corporation). As shown in Figure 4C, while about 70% of primary cells exhibited and arrested at G0/G1phases, the percentage of K4D and K4DT cells in G0/G1 phases was about 50% less. The representative histogram of wild type, K4D, and K4DT cells is shown in Figure 4C. The ratio of S and G2/M phases in K4D and K4DT cells was about 20% higher than in primary cells, which had around 15% S and G2/M phase cells. The results of the cell cycle analysis are summarized in Table 2, and the statistical difference was evaluated. As shown in Table 2, a decreased ratio of G0/G1 and increased ratio of S phase and G2/M phase cells were detected. From the results of cell cycle analysis, we concluded that K4DT cells showed accelerated turnover of the cell cycle due to the expression of CDK4 and Cyclin D1.

**TABLE 2:** Cell cycle analysis of wild type, K4D and K4DT cells.

Cell line	Cell cycle phase			
	G0/G1	S	G2/M	Debris
Wild type	70.7 ± 1.4	3.4 ± 0.1	15.5 ± 0.9	61.5 ± 4.0
K4D	48.8 ± 0.8**	5.4 ± 0.2**	19.7 ± 0.3**	27.2 ± 1.4**
K4DT	51.0 ± 0.5**	6.3 ± 0.1**	21.0 ± 0.2**	26.1 ± 1.2**

**^∗∗^p < 0.01.**

### Real-Time PCR Detection of Androgen Receptor Expression

In our previous study, we showed that expression of the androgen receptor in DPCs was suppressed after several passages, even in primary culture. Kwack et al. (2010) reported that the mRNA expression level of the androgen receptor dramatically decreases after passage six compared to the original cell culture. To determine whether our immortalized DPCs exhibited suppression of the androgen receptor, we used real-time PCR to detect the endogenous expression level of the androgen receptor. As shown in Figure 4D, total RNA from normal prostate tissue was used as a positive control. The human embryonic fibroblast line HE16 was used as a negative control. When the expression level of the androgen receptor in wild type DPCs at passage eight was set to 1.0, the relative expression level in HE16 cells was close to 1.0, while the relative expression level in normal prostate-derived RNA was more than 42. The expression level in immortalized K4DT DPCs at passage 12 was almost the same as that in primary DPCs and HE16 fibroblasts. Amplification plots of AR was shown in Supplementary Figure S7. These data indicated that primary DPCs exhibited suppression of the androgen receptor, probably due to the several passages performed during manufacturing. From these data, we concluded that our established immortalized DPCs are negative for androgen receptor expression.

### Retroviral Introduction of the Androgen Receptor Into Immortalized DPCs

**FIGURE 5:**
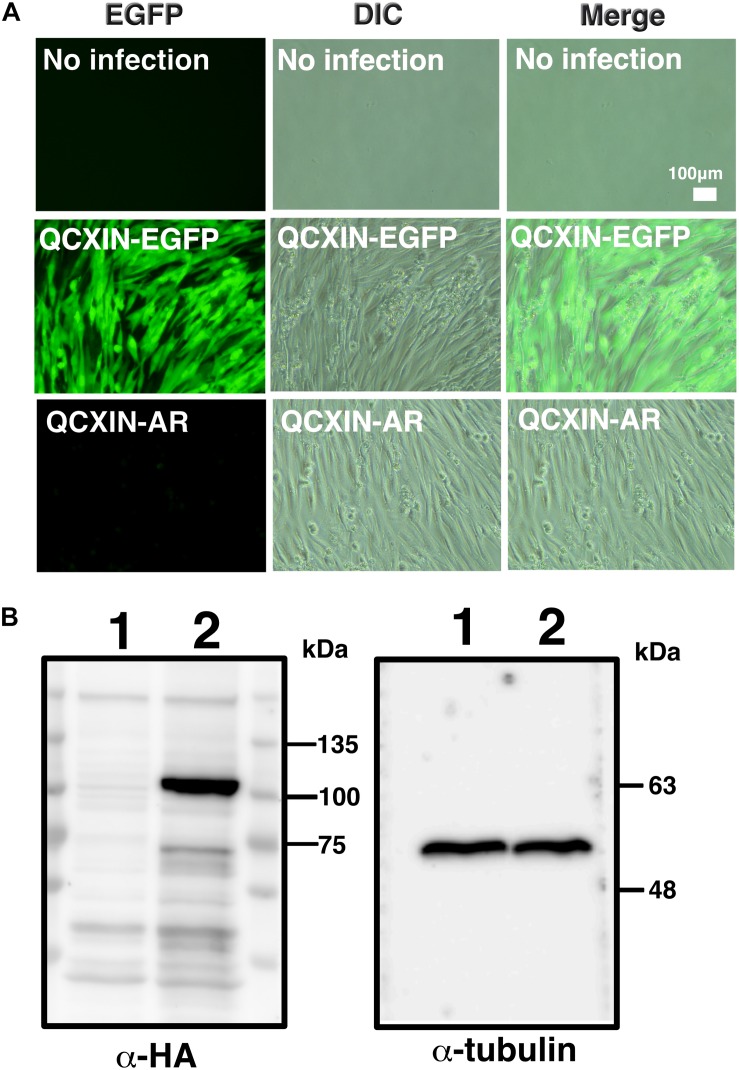
Retroviral introduction of the androgen receptor into immortalized DPCs with K4DT. **(A)** G418 selection of wild type DPCs (no infection), QCXIN-EGFP-infected DPCs, and QCXIN-AR-infected DPCs. Note that wild type (no infection) cells exhibited complete cell death upon addition of 700 μg/mL G418. **(B)** Western blot analysis of QCXIN-EGFP (lane 1) and QCXIN-AR (lane 2) probed with HA protein tag antibody (Left panel), and α-tubulin antibody (Right panel). Note that the HA positive band was observed at around 100 kDa in the left panel.

As shown in Figure 4D, original DPCs and immortalized DPCs showed low expression of the androgen receptor. To estimate the efficiency of gene introduction via retrovirus, retroviruses expressing EGFP were used to infect K4DT cells. As shown in the upper panels of Supplementary Figure S1, a small percentage of K4DT cells were infected. There was no obvious cell toxicity after infection with androgen receptor-expressing retroviruses (Supplementary Figure S1, lower panels), and we did not detect any morphological changes in infected cells. The efficiency of infection was not as high as described in the previous section. Due to the presence of a neomycin-resistance gene downstream of the EGFP or androgen receptor-expressing retrovirus (QCXIN retrovirus, Takara Bio, Shiga, Japan), we were able to perform selection using G418 antibiotics to purify the EGFP or androgen receptor-expressing cells. As shown in Figure 5A, DPCs expressing K4DT with no additional infection showed complete cell death after G418 selection (700 μg/mL) (Figure 5A, upper panels). However, the K4DT DPCs successfully infected by QCXIN-EGFP or QCXIN-AR showed resistance to G418 selection (Figure 5A, middle and lower panels). Furthermore, after infection with QCXIN-EGFP, the surviving cells selected for by administration of 700 μg/mL of G418 were all positive for EGFP expression (Figure 5A, middle panel), indicating that antibiotic selection worked properly. To verify androgen receptor expression in DPCs with K4DT, we carried out western blotting using an HA antibody. As shown in Figure 5B, although α-tubulin did not show any difference in signal intensity (Figure 5B, right panel and Supplementary Figure S4), DPCs infected with QCXIN-AR showed a specific signal at 100 kDa in the western blot for HA antibody (Figure 5B, Left panel, lane 2 and Supplementary Figure S4). From these data, we concluded that we successfully established K4DT DPCs expressing the androgen receptor.

### Visualization of Cellular Localization of the Androgen Receptor

**FIGURE 6:**
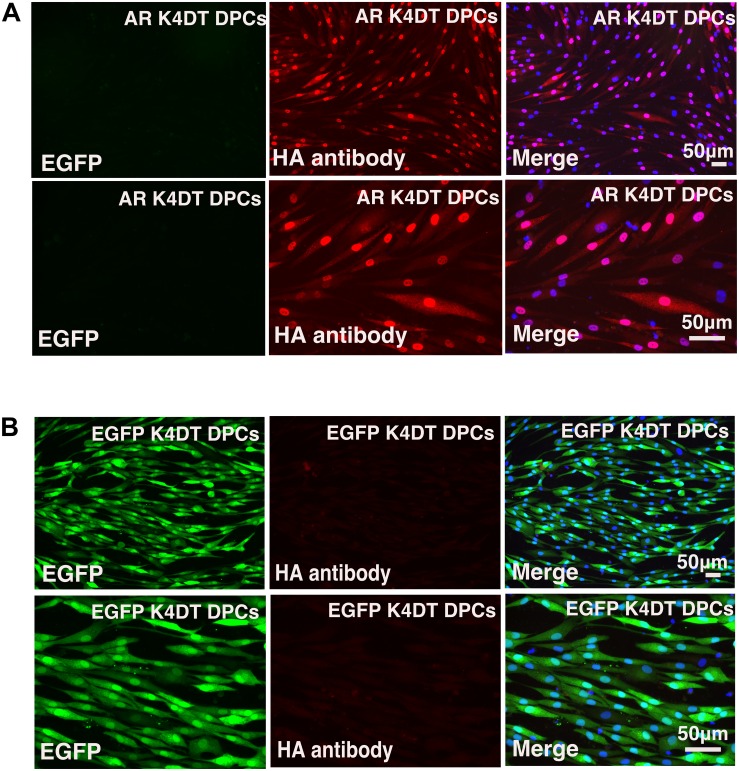
Immunohistochemical detection of the androgen receptor using an HA antibody. **(A)** Immunohistochemical detection of androgen receptor-expressing DPCs immortalized with expression of mutant CDK4, Cyclin D, and TERT. EGFP (left panels), androgen receptor detected using an HA antibody (middle panels), and a merged image with EGFP, androgen receptor staining, and nuclear counterstaining with DAPI (Right panels). Upper panels are low magnification. Lower panels are high magnification. Note that nuclear and cytoplasmic localization of androgen receptor detected with HA antibody. **(B)** Detection of fluorescence in immortalized DPCs expressing QCXIN-EGFP. EGFP fluorescence (Right panels), HA antibody staining (middle panels), and a merged image with EGFP, HA antibody staining, and nuclear counterstaining with DAPI (Right panels). Lower panels are high magnification.

After stable introduction of the androgen receptor into K4DT DPCs, we detected the cellular localization of the androgen receptor. As shown in Figure 6B, K4DT DPCs with QCXIN-EGFP (control) did not show any reactivity with the HA antibody (Figure 6B, Middle upper panel), indicating the specific binding of the antibodies. Interestingly, K4DT DPCs with QCXIN-AR showed positive staining in the cytoplasm and nuclear (Figure 6A, Upper middle panel). As shown in the merged picture with 4′,6-diamidino-2-phenylindole (DAPI) (upper right panel of Figure 6A), staining for the androgen receptor via the HA tag revealed cytoplasmic and nuclear localization, in agreement with the previous manuscript (Fang et al., 1996). From these results, we concluded that we successfully established immortalized DPCs with constant expression of the androgen receptor.

### Cellular Distribution of F-Actin

**FIGURE 7:**
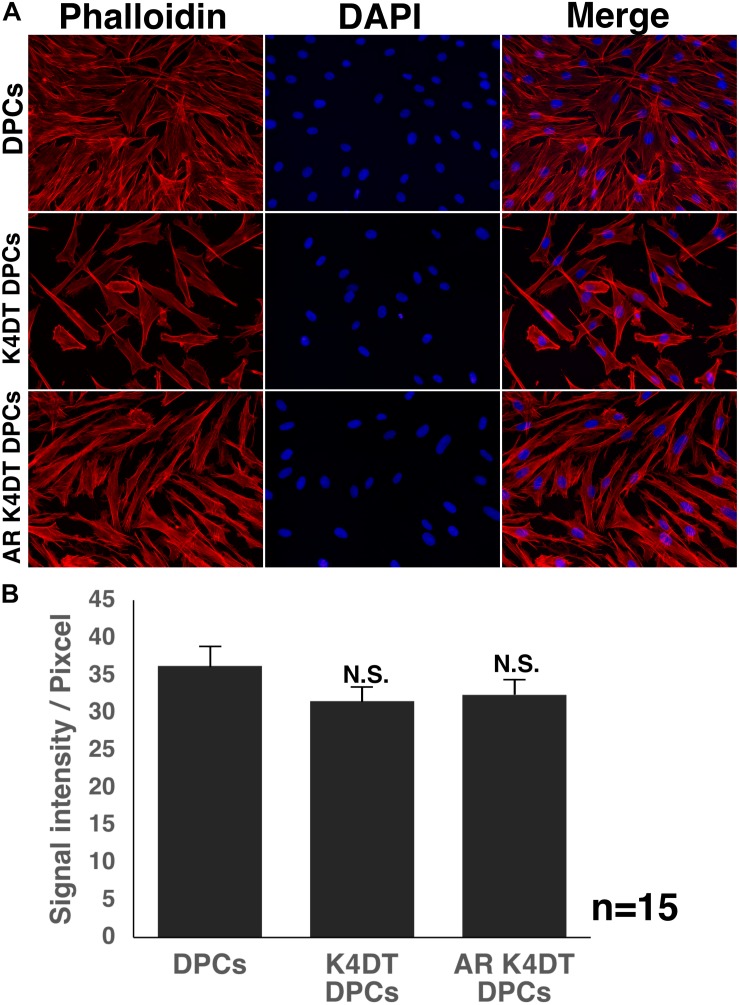
Detection of cytoskeletal F-actin in wild type, K4DT, AR expressing K4DT DPCs. **(A)** Staining feature of wild type, K4DT, and AR expressing K4DT DPCs. Staining with phalloidin, DAPI, and merged pictures were presented. **(B)** The quantitation of fluorescence intensity from the cytoplasm of 15 randomly selected cells. Average and standard error were shown in the graph. No significant difference.

The cytoskeletal F-actin has critical role for the cell migration, and cell division and proliferation (Debacq-Chainiaux et al., 2009). We detected the cellular distribution of F-actin among wild type, K4DT, androgen receptor (AR) expressing K4DT DPCs. In the fluorescence images, we could not observe significant difference of F-actin in these three types of cells (Figure 7A). Furthermore, we measured the fluorescence intensity of randomly selected 15 cytoplasm (Supplementary Figure S5). As shown in Figure 7B, we did not observe any statistical difference among wild type, K4DT, and AR expressing K4DT DPCs.

### Expression of AR in K4DT DPCs Induces the Expression of Downstream Gene, Dkk1

Before and after the introduction of AR, we also exposed 100 nM of dihydrotestosterone to the cells. After the exposure, we detected the expression of Dkk1, which is known to locate the downstream of gene network of testosterone signaling. We used Taqman probe method for the accurate quantitation of Dkk1. In Figure 9A, we showed the amplification plots of Dkk1 mRNA in AR expressing DPCs and EGFP expressing DPCs with and without treatment of 100 nM of dihydrotestosterone. From the amplification curve, we showed the relative expression of Dkk1 has dramatically elevated after the exogenous introduction of AR (Figure 9B and Supplementary Figure S9). Interestingly, expression level was around 70% after the treatment of dihydrotestosterone.

**FIGURE 8:**
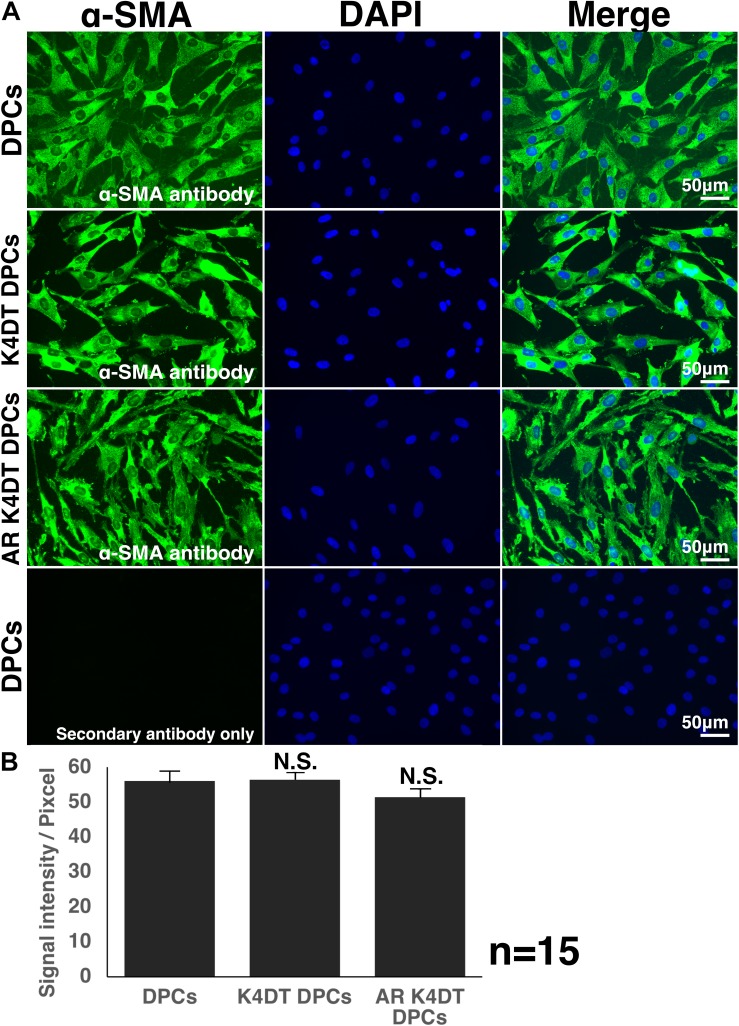
Detection of α-smooth muscle actin (SMA) in wild type, K4DT, AR expressing K4DT DPCs. **(A)** Staining feature of wild type, K4DT, and AR expressing K4DT DPCs. Note that there is no staining signal of SMA in the absence of primary antibody. Staining signals of SMA, DAPI, and Merged pictures were shown. **(B)** The quantitation of fluorescence intensity from the cytoplasm of 15 randomly selected cells. Average and standard error were shown in the graph. No significant difference.

**FIGURE 9:**
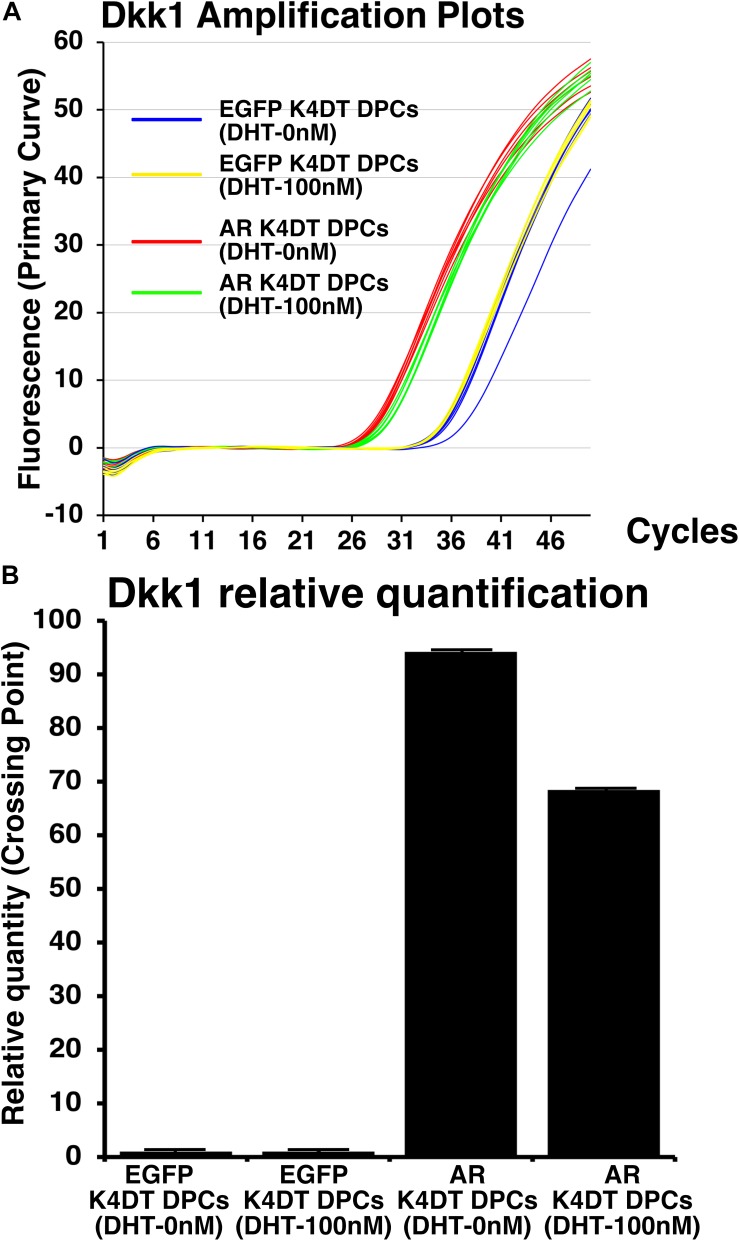
Detection of Dkk1 expression before and after the exogeneous introduction of androgen receptor (AR), and absence and presence of dihydrotestosterone (DHT). **(A)** Amplification plots of endogenous Dkk1 in EGFP expressing K4DT DPCs and AR expressing K4DT DPCs. **(B)** The quantitation of Dkk1 expression with ΔΔ Ct method. The relative quantitation value of samples were shown in the graph. When one of the sample of EGFP expressing K4DT DPCs was set as 1.0, the expression level of samples were calculated after the adjustment with glyceraldehyde-3-phosphate dehydrogenase (GAPDH). Number of samples is 6 for each group.

Furthermore, Inui et al. reported that TGFβ1 is upregulated by androgen in DPCs of AGA, suggesting that TGFβ1 is the potential downstream of testosterone signaling pathway (Inui et al., 2002). To evaluate this possibility, we detected the expression level of TGFβ1 in EGFP expressing DPCs, and AR expressing DPCs, under the absence and presence of DHT. As shown in Supplementary Figures S8A,B, although we observed marginal increase after the introduction of AR into K4DT DPCs, but its upregulation was not so evident. Furthermore, the expression difference of TGFβ1 was not apparent between absence and presence of AR, when it compared with that of Dkk1. Amplification plots of GAPDH for TGFβ1 showed almost no difference among the samples, indicating that slight increase of TGFβ1 expression is not due to the sample quality (Supplementary Figure S10). From this situation, we explained that TGFβ1 might be mainly regulated by various types of signaling pathways, although testosterone signaling pathway would be one of the factors of them.

### Nuclear Translocation Is Accelerated by the Treatment of Dihydrotestosterone

**FIGURE 10:**
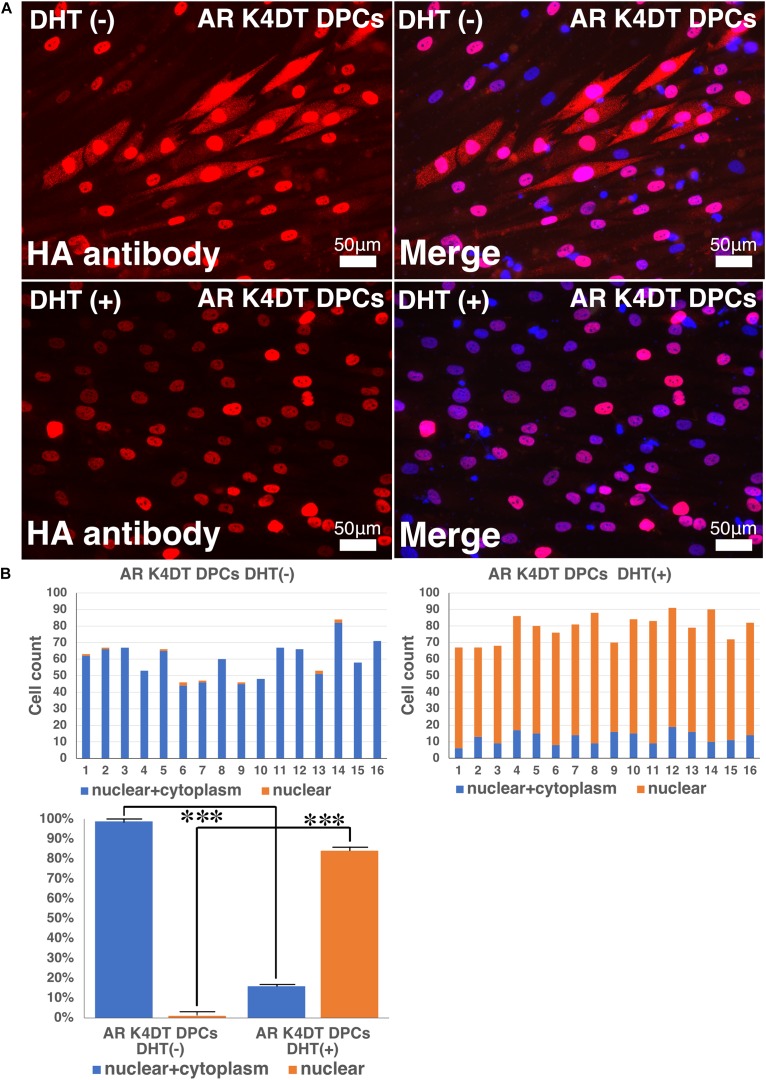
Detection of nuclear translocation of androgen receptor (AR) under absence and presence of dihydrotestosterone (DHT). **(A)** Representative staining feature of AR K4DT DPCs with and without DHT. Note that under the presence of DHT, the localization of AR in the cytoplasm is almost negative, and AR localize within the nuclear. **(B)** Count of cells, which positive nuclear and cytoplasm (blue bar) and cells, which positive only within the nuclear (orange bar). Left side, results from 16 images before DHT treatment. Right side, results from 16 images after DHT treatment. Bottom panel, percentage of cells, which positive both nuclear and cytoplasm (blue bar) and percentage of cells, which positive only within the nuclear (orange bar). The statistical significance more than 0.1% were shown with three stars. *N* = 16 for each group.

To detect the functionality of exogenous introduced AR, we detected localization of nuclear and cytoplasm localization of AR with HA tag antibody. As shown in Figure 10A, AR expressing K4DT DPCs showed pale positive staining in the cytoplasm and nuclear staining, before DHT treatment. However, to be noted, the pale positive staining in the cytoplasm has disappeared (Figure 10A, lower left), and the localization of AR becomes only limited within the nuclear (Figure 10A, lower right). The incidence of the nuclear + cytoplasm cell becomes significantly lower after the DHT treatment (Figure 10B). Furthermore, the incidence of cells which showed the positive staining only within the nuclear significantly increased after the DHT treatment (Figure 10B). From these observations, we concluded that our introduced AR with HA tag works properly against DHT ligand treatment.

### Anti-testosterone Reagent, MDV3100, Partially Inhibit the Nuclear Translocation of AR

**FIGURE 11:**
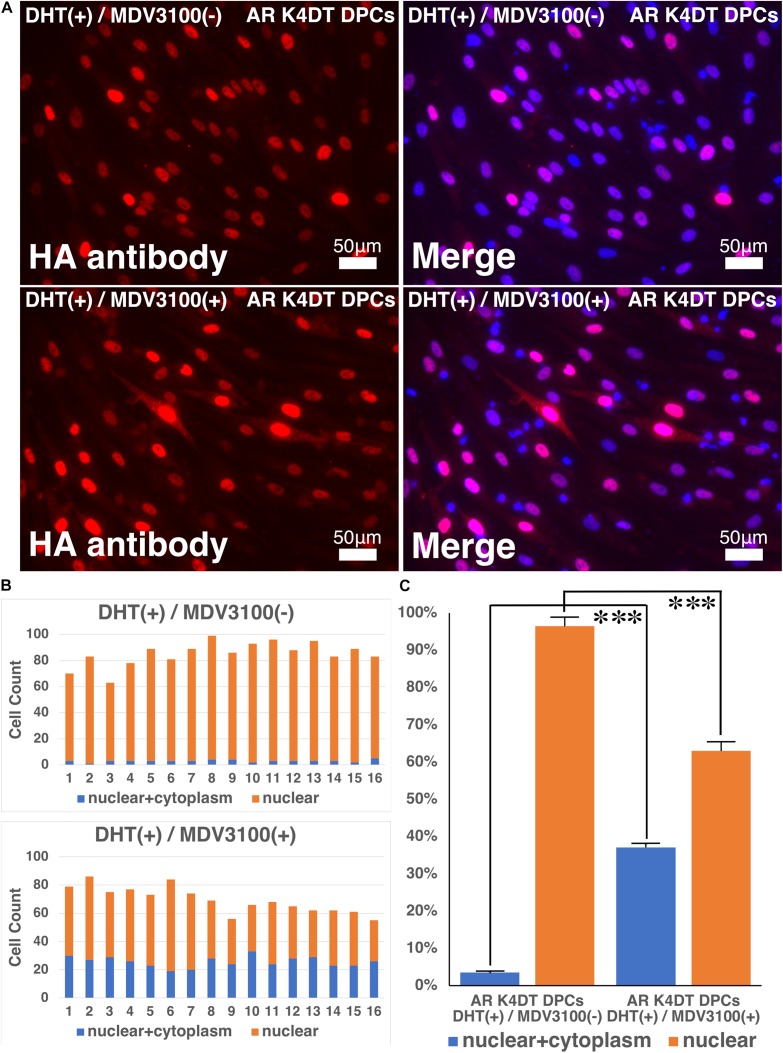
Nuclear translocation of AR by DHT is partially inhibited by MVD3100 in AR expressing K4DT DPCs. **(A)** Representative staining feature of AR with HA tag antibody after the DHT treatment, under the absence and presence of MDV3100. **(B)** Upper panel, Count of cells, which positive nuclear and cytoplasm (blue bar) and cells, which positive only within the nuclear (orange bar) under the absence of MDV3100. Lower panel, Count of cells, which positive nuclear and cytoplasm (blue bar) and cells, which positive only within the nuclear (orange bar) under the existence of MDV3100. **(C)** Percentage of cells, which positive both nuclear and cytoplasm (blue bar) and percentage of cells, which positive only within the nuclear (orange bar). Comparison between absence of presence of MDV3100. The statistical significance more than 0.1% were shown with three stars. *N* = 16 for each group.

In previous section, we showed that our established AR expressing K4DT DPCs detect the nuclear translocation of AR with HA antibody after the DHT treatment. These results guided us to build the hypothesis that our established cell might be useful to detect the anti-testosterone effect. We evaluated the effects of MDV3100, which is distributed as anti-testosterone compound, Enzalutamide in clinical stage of prostate cancer treatment. MDV3100 (Enzalutamide) is authorized as the anti-prostate cancer drug (Gibbons et al., 2015), and inhibits nuclear translocation of AR, and coactivator recruitment (Tran et al., 2009). Based on these clinical data, we exposed the 10 μM of MDV3100 to the cell culture medium under the existence of 100 nM DHT. As shown in Figure 11, although treatment of DHT completely erased the positive signal of the cytoplasm as upper right panel of Figure 11A, addition of MDV3100 showed existence of pale positive cells for their cytoplasm (Figure 11A, lower right). For the accurate evaluation, we counted the cell count of cells, which both positive for cytoplasm and nuclear (blue bar in Figure 11B), or cells, which only positive within the nuclear (orange bar in Figure 11B). Based on the counting data, we summarized the percentage of cells, which both positive for cytoplasm and nuclear (blue bar) or cells, which only positive within the nuclear (orange bar), absence and presence of MVD3100, in Figure 11C. As the results of statistical difference, we concluded that the MDV3100 treatment significantly increases the cell incidence which positive both for nuclear and cytoplasm (Figure 11C, blue bar). Furthermore, MDV3100 treatment significantly decreased the incidence of cells, which only positive nuclear (Figure 11C, orange bar). From these data, we concluded that treatment of MDV3100 partially inhibit the translocation of AR from cytoplasm to nuclear.

## Discussion

In this study, we established the first immortalized human follicle dermal papilla cells expressing mutant CDK4, Cyclin D1, and TERT, and we showed that immortalized DPCs retained the characteristics of primary cells as well as an intact chromosomal karyotype. In the previous manuscript, DPCs with SV40 were reported (Kim et al., 2003). However, as shown in swine embryonic fibroblasts, even at passage 8 these cells already exhibited polyploid abnormalities at a rate of 16.4%, indicating that SV40-expressing cells frequently experience chromosomal abnormalities (Fukuda et al., 2012a).

The combined expression of mutant CDK4, Cyclin D1, and TERT enables us to efficiently immortalize various cell types, as mentioned previously. The basic principle of this K4DT immortalization method is bypassing the negative feedback signal from the senescence protein p16 (Fukuda et al., 2018). Once cells reach the senescence phase, p16 accumulates in the cells (Shiomi et al., 2011). p16 proteins bind to CDK4 and negatively regulate the kinase activity of the CDK4-Cyclin D1 complex, resulting in a slowdown of cell cycle turnover. The R24C mutant of CDK4 is resistant to negative regulation via p16 due to an amino acid alteration at the p16 binding site. Co-expression of Cyclin D1 forms a functional enzymatic complex, resulting in continuous cell growth. However, expression of mutant CDK4 and Cyclin D1 is not enough, since these genes are not able to prevent shortening of the telomere sequence at the end of chromosomes. The addition of TERT expression allows for extension of the telomere sequence, resulting in immortalization. We previously reported that this K4DT method can be applied to various cell types, such as fibroblasts (Katayama et al., 2016) and intestinal epithelial cells (Kuroda et al., 2015b). In this study, we showed that expression of mutant CDK4, Cyclin D, and TERT allows us to efficiently establish immortalized cells from human DPCs.

We showed that immortalized K4DT cells retain the original nature of primary cells. We observed positive staining for alkaline phosphatase, a surrogate marker, suggesting that our established K4DT DPCs retained the original nature of the primary cells. In agreement with this result, a previous study showed that K4DT cells derived from the human skeletal muscle cells of patients with myotonic dystrophy type 1 (DM1) retained the original pathogenic condition, such as the abnormal splicing of exon 11 of BIN1 (bridging integrator-1) and exon 5 of MBNL1 (muscle-specific splicing factors muscle blind-like protein 1) (Pantic et al., 2016). However, for a more precise classification of the nature of the original cells, global transcriptome analysis would be necessary. As the supportive evidences of original nature, we detected the cellular distribution of F-actin and SMA (Figure 8 and Supplementary Figure S6). We also detected the fluorescence intensity of F-actin and SMA in randomly selected area, we did not detect any difference among wild type, K4DT, and AR expressing K4DT DPCs. These data suggest that K4DT method would be more advantageous to keep the nature of the original cells.

Through chromosome analysis, we observed that 100% of cells exhibited perfect normal condition, 46 + XY. However, in the G-banding analysis, one sample out of 20 mitotic cells possessed an abnormality in chromosome 1. We previously showed that around 6.5% of cells exhibited abnormal chromosome patterns even in normal swine fibroblasts (Fukuda et al., 2012a). Therefore, approximately 5% of observed chromosome abnormalities can be attributed to an artifact caused by sample preparation for chromosome analysis.

Through senescence-associated β-gal staining, we observed that several K4D cells showed intense staining, but the majority of cells did not. Though not confirmed, the estimated efficiency of gene introduction was around 80–85% based on the results from enhanced green fluorescence protein (EGFP)-expressing lentivirus (Figure 1B). Since efficient cell growth requires double infection with mutant CDK4 and Cyclin D1-expressing lentiviruses, an estimated 64% of cells would be doubly-infected, while the rest (∼36%) would have a single transgene, mutant CDK4 or Cyclin D1. There is a possibility that the SA-positive cells described above are cells infected with a single transgene.

When considering the biology of DPCs and their relationship with AGA, we must note the importance of the androgen receptor, since DHT is classified as one of the major causes of the progression of AGA. Hormone dependency must be implemented when using cultured DPCs as a research tool for studying AGA (Kwack et al., 2008, 2010). Upon recognizing this issue, we determined the endogenous expression level of the androgen receptor in immortalized DPCs, as shown in Figure 4D. Based on the results in Figure 4D, we concluded that the endogenous expression level of the androgen receptor was already suppressed during the cell culture period of the provider, which is good agreement with the previous finding that expression level of androgen receptor was strongly suppressed in primary rat derived DPCs. In brief, Kwack et al. (2008) showed that the expression level of the androgen receptor gene dramatically decreased after passage 6. Although the detailed mechanism for the gene silencing of the androgen receptor is not understood, we decided to introduce the androgen receptor gene under an artificial promoter. Furthermore, we introduced an HA protein tag to facilitate detection of the introduced androgen receptor. Western blot analysis revealed high expression of the androgen receptor via its expression cassette in our established immortalized DPCs. Furthermore, we also detected beautiful localization of the androgen receptor in the nuclear and cytoplasm of our established cells. Furthermore, we showed that downstream gene, Dkk1, is highly activated after the introduction of AR. The elevated mRNA level of Dkk1 indicate that our established AR expressing K4DT DPCs involves the gene network related to testosterone. Interestingly, we detected the expression level of TGFβ, which previously identified as elevated gene after the DHT treatment. However, we did not observe any significant change in the expression level of TGFβ even after the expression of AR and existence of DHT. The biological significance of TGFβ in DPCs would be necessary to be addressed in further investigation.

Furthermore, owing to the introduction of an HA protein tag, we successfully detected the translocation of introduced AR into the nucleus after the DHT treatment. The sensitivity of the high affinity HA antibody is so high that a cytoplasm-to-nucleus transition could be effectively detected by this system. Furthermore, we showed that MDV3100 (Enzalutamide) partially inhibits the nuclear translocation of AR. MDV3100 (Enzalutamide) is authorized drug for prostate cancer, and has anti-testosterone activity. To develop more specific and efficient anti-testosterone drugs, various types of compounds are under development. As one of the candidates, CH5137291 has reported to have more strong effect to inhibit the nuclear translocation of AR. Although CH513791 is not distributed yet in the market, we are planning to test the effect of CH513791 to AR translocation as next study. The established immortalized DPC line with androgen receptor expression described in this study is a beneficial tool for the screening of anti-testosterone compounds, which would be useful for the prevention of AGA and other testosterone-related diseases, such as prostatic hypertrophy and prostate cancer. We plan to share our established DPCs with scientists worldwide to facilitate further research using this innovative cell line.

## Data Availability Statement

The data sets used and/or analyzed during the current study are available from the corresponding author on reasonable request.

## Author Contributions

TF, KT, ST, AO, and TE did the experiments. TF, KN, and TK contributed the design of the experiments. TF and KT wrote the manuscript. TK provided the experimental materials.

## Conflict of Interest

The authors declare that the research was conducted in the absence of any commercial or financial relationships that could be construed as a potential conflict of interest.

## Supplementary Material

FIGURE S1 Detection of potential cell morphological changes and fluorescence of immortalized DPCs expressing QCXIN-EGFP and QCXIN-AR before G418 selection. Immortalized DPCs infected with QCXIN-EGFP (upper panels). Immortalized DPCs infected with QCXIN-AR (lower panels).

FIGURE S2 Full length gel photos for Figure 2A. Upper panel, detected gel image of CDK4 and Cyclin D. Lower panel, detected gel image of TERT and TSC2.

FIGURE S3 Full length blot photo for Figure 2B. Upper penal, detected blot image of CDK4. Middle panel, detected blot image of Cyclin D. Lower panel, detected image of alpha-tubulin. The corresponding area of Figures are marked by the rectangles.

FIGURE S4 Full length blot photo for Figure 5B. Upper panel, detected blot image of HA antibody. Lower panel, detected blot image of alpha-tubulin. The corresponding area of Figures are marked by the rectangles.

FIGURE S5 F-actin staining of measurement of fluorescence intensity of the 15 area. The area of the measurement of fluorescence intensity in wild type DPCs, K4DT DPCs, AR expressing DPCs were shown with white rectangles.

FIGURE S6 Immunostaining of α-smooth muscle actin (SMA) of wild type, K4DT, and AR expressing K4DT DPCs. The area of the measurement of fluorescence intensity in wild type DPCs, K4DT DPCs, AR expressing DPCs were shown with white rectangles.

FIGURE S7 Amplification plots of androgen receptor (AR) with real time PCR analysis. Expression AR in wild type DPCs, K4DT DPCs, HE16, human normal prostate derived RNA were evaluated.

FIGURE S8 Amplification plots of TGFβ1 with real time PCR analysis. **(A)** Amplification plots of TGFβ1 in K4DT DPCs and AR expressing K4DT AR DPCs with and without dihydrotestosterone. **(B)** The quantitation of TGFβ1 expression with ΔΔ Ct method.

FIGURE S9 Amplification plots of glyceraldehyde-3-phosphate dehydrogenase (GAPDH) with real time PCR method for the quantitation of Dkk1.

FIGURE S10 Amplification plots of glyceraldehyde-3-phosphate dehydrogenase (GAPDH) with real time PCR method for the quantitation of TGFβ1.

## Abbreviations

AGA: Androgenetic alopecia
DPCs: dermal papilla cells
CDK: 4cyclin-dependent kinase 4
TERT: telomerase reverse transcriptase
DHT: dihydrotestosterone
TGFβ: transforming growth factor β
Dkk1: Dickkopf-related protein 1
DAPI: 4′,6-diamidino-2-phenylindole
SA-β-Gal: senescence-associated β-galactosidase
AP: alkaline phosphatase.
