# Supplementary figures and images for: Human Derived Immortalized Dermal Papilla Cells With a Constant Expression of Testosterone Receptor
Source: Front Cell Dev Biol. 2020 Mar 18;8:157. doi: 10.3389/fcell.2020.00157 (PMC7109449; doi:10.3389/fcell.2020.00157)

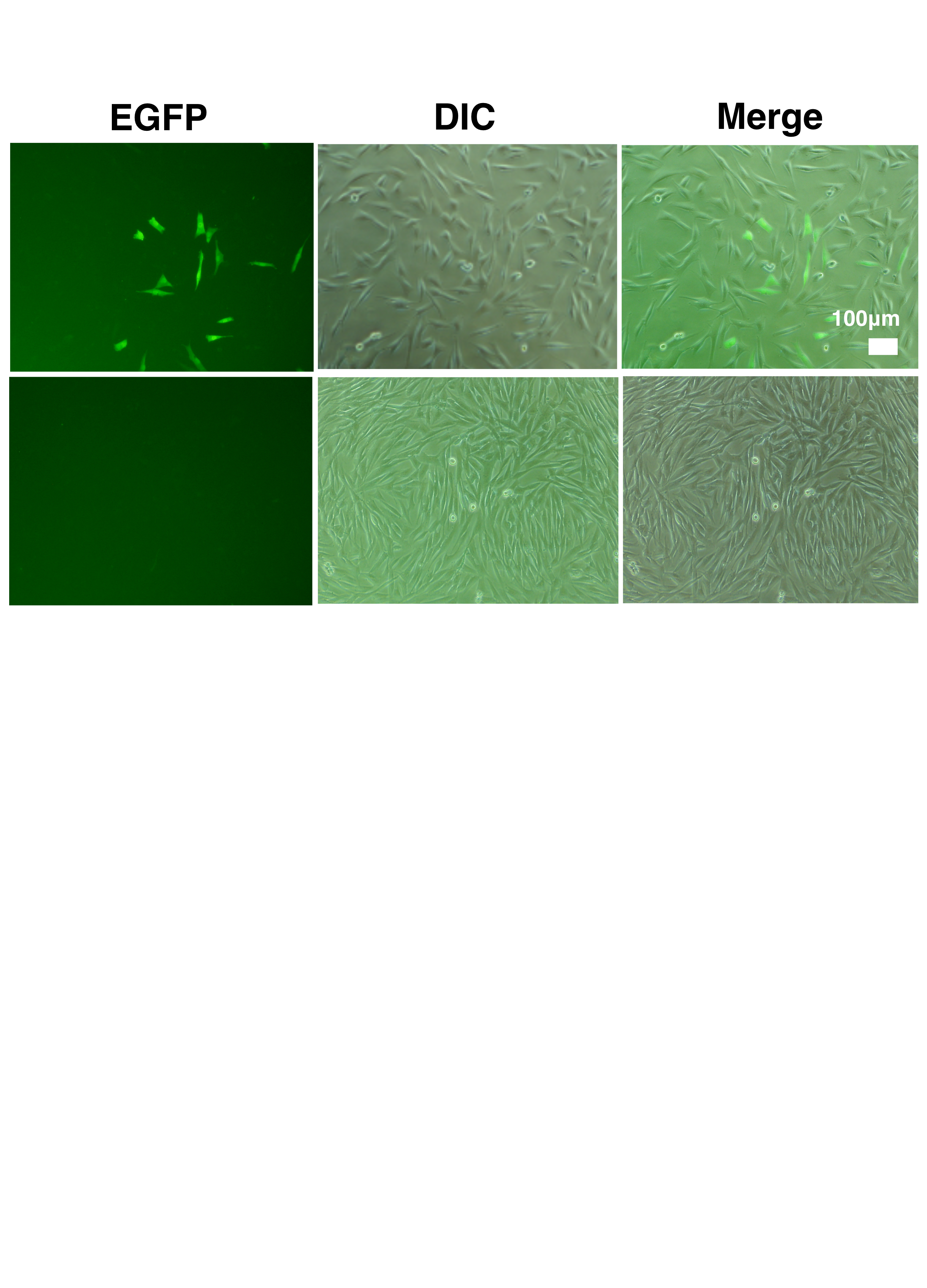

Supplement: FIGURE S1 — Detection of potential cell morphological changes and fluorescence of immortalized DPCs expressing QCXIN-EGFP and QCXIN-AR before G418 selection. Immortalized DPCs infected with QCXIN-EGFP (upper panels). Immortalized DPCs infected with QCXIN-AR (lower panels). [file Image_1.TIFF]

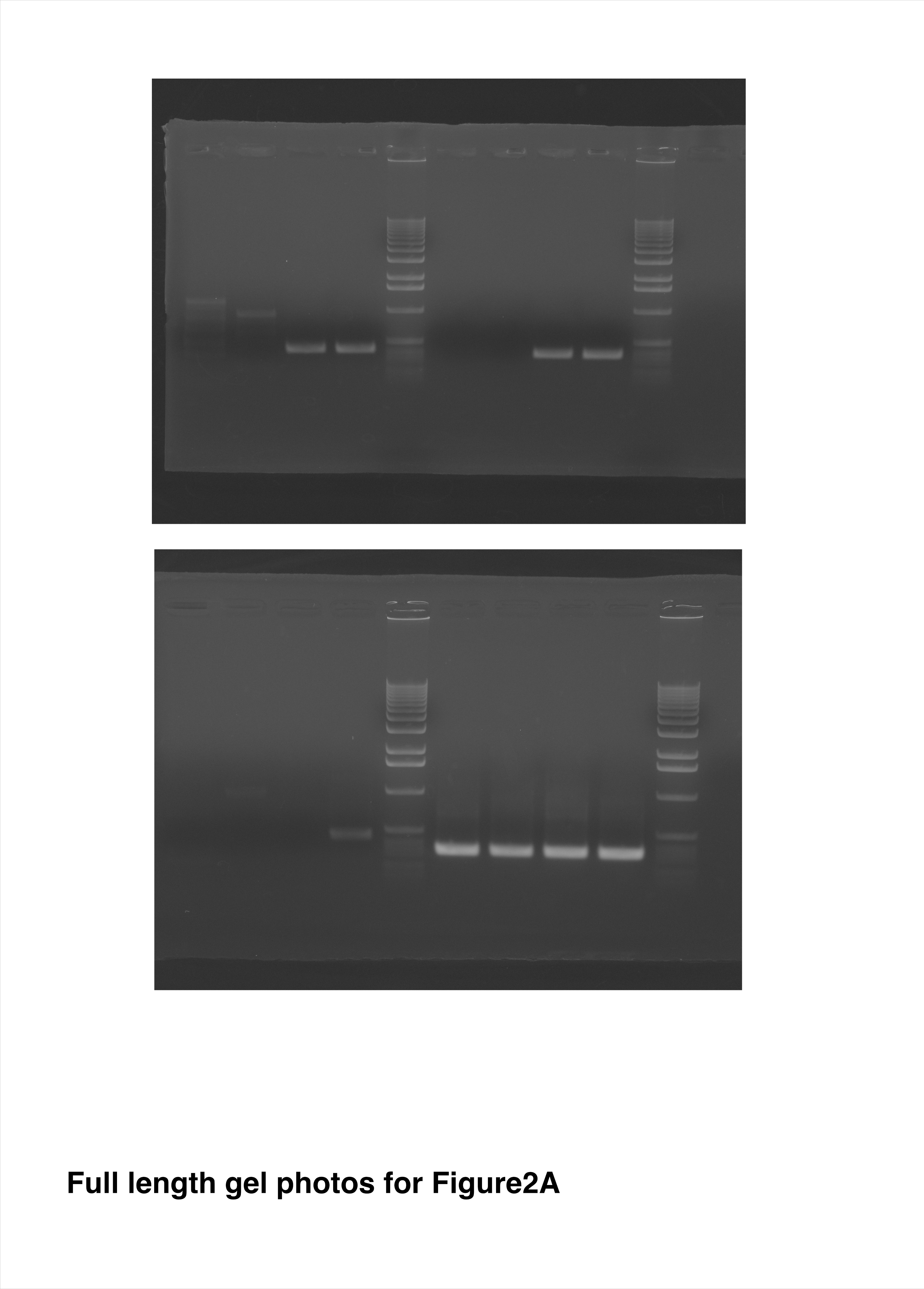

Supplement: FIGURE S2 — Full length gel photos for Figure 2A. Upper panel, detected gel image of CDK4 and Cyclin D. Lower panel, detected gel image of TERT and TSC2. [file Image_2.TIFF]

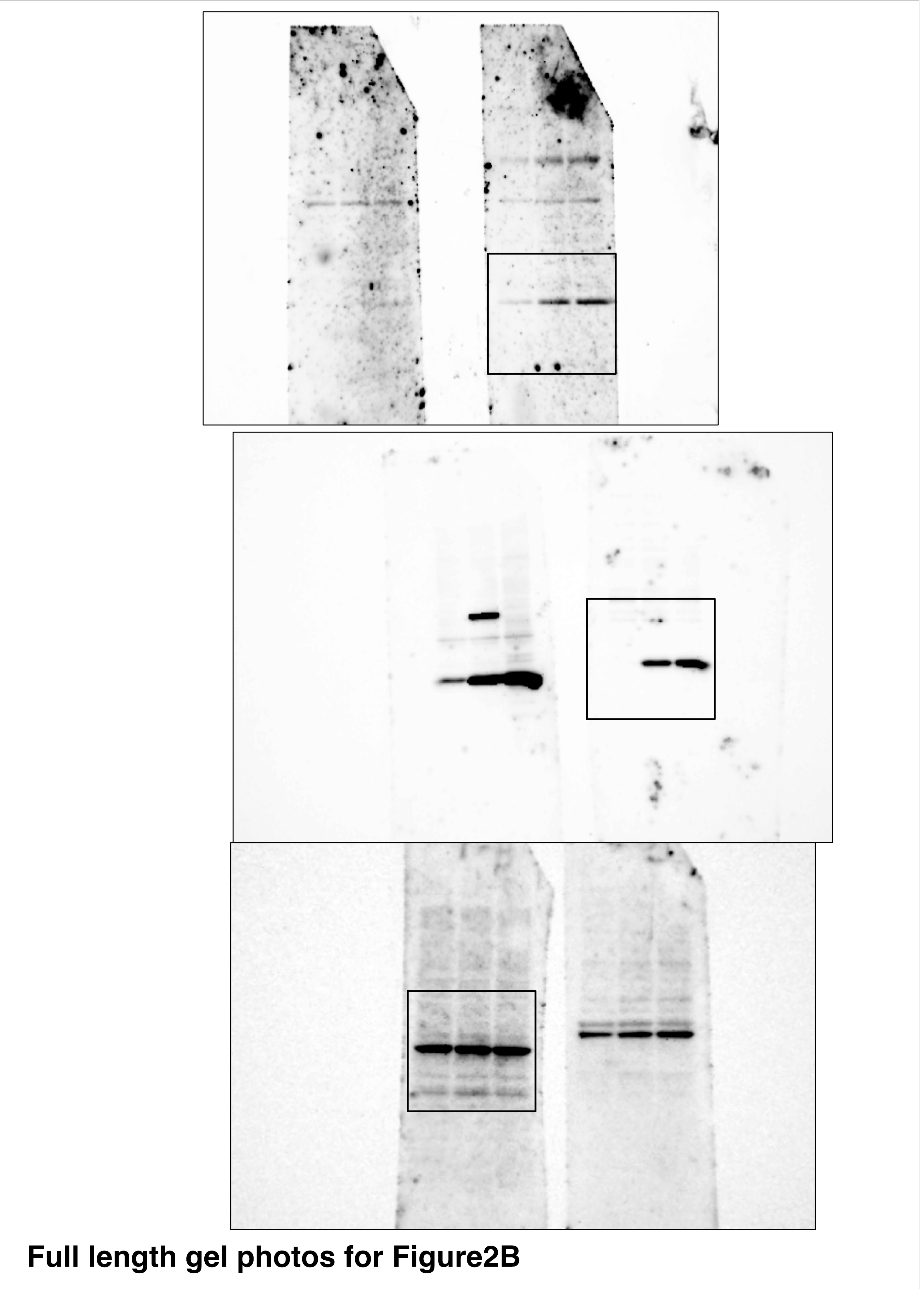

Supplement: FIGURE S3 — Full length blot photo for Figure 2B. Upper penal, detected blot image of CDK4. Middle panel, detected blot image of Cyclin D. Lower panel, detected image of alpha-tubulin. The corresponding area of Figures are marked by the rectangles. [file Image_3.TIFF]

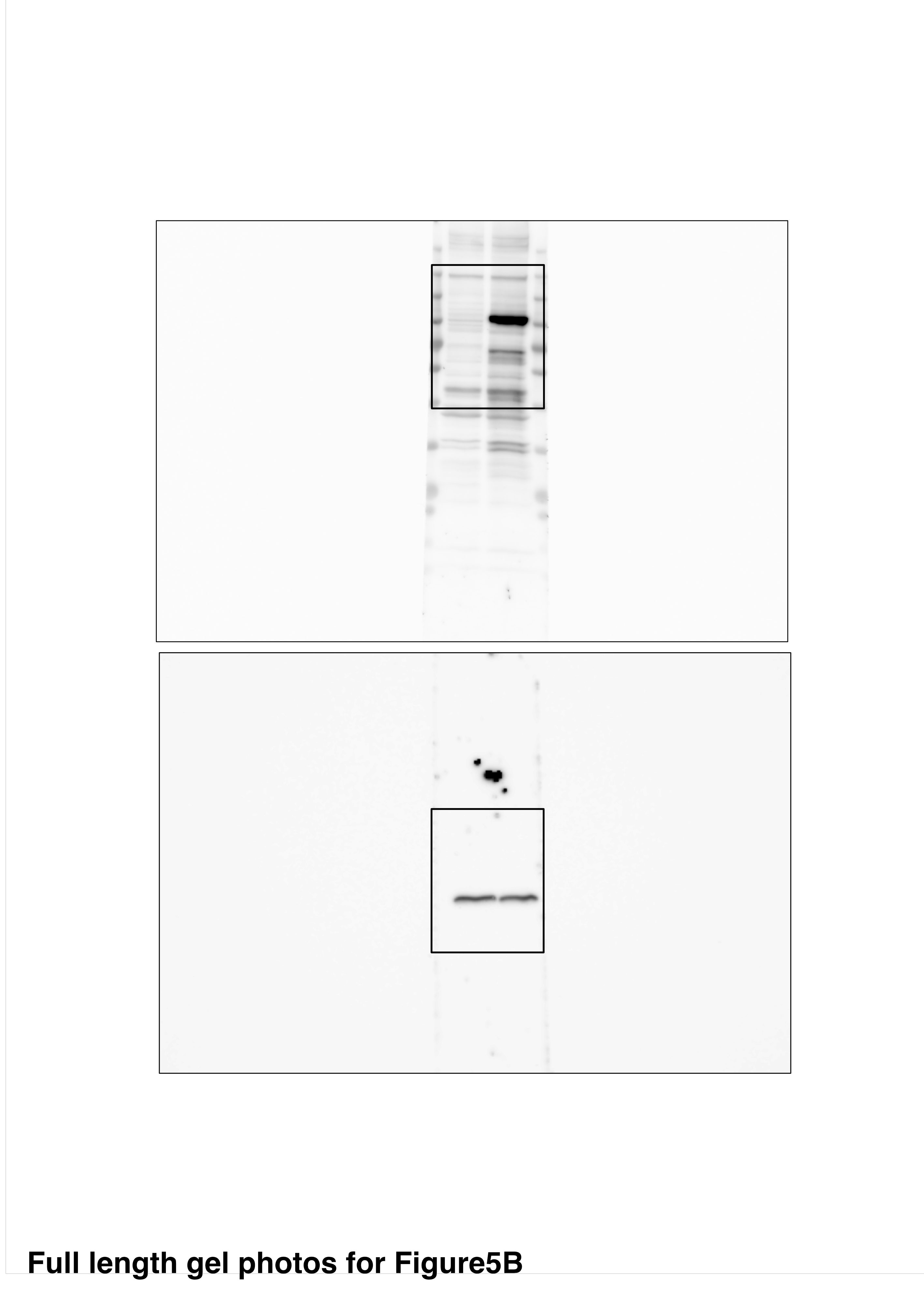

Supplement: FIGURE S4 — Full length blot photo for Figure 5B. Upper panel, detected blot image of HA antibody. Lower panel, detected blot image of alpha-tubulin. The corresponding area of Figures are marked by the rectangles. [file Image_4.TIFF]

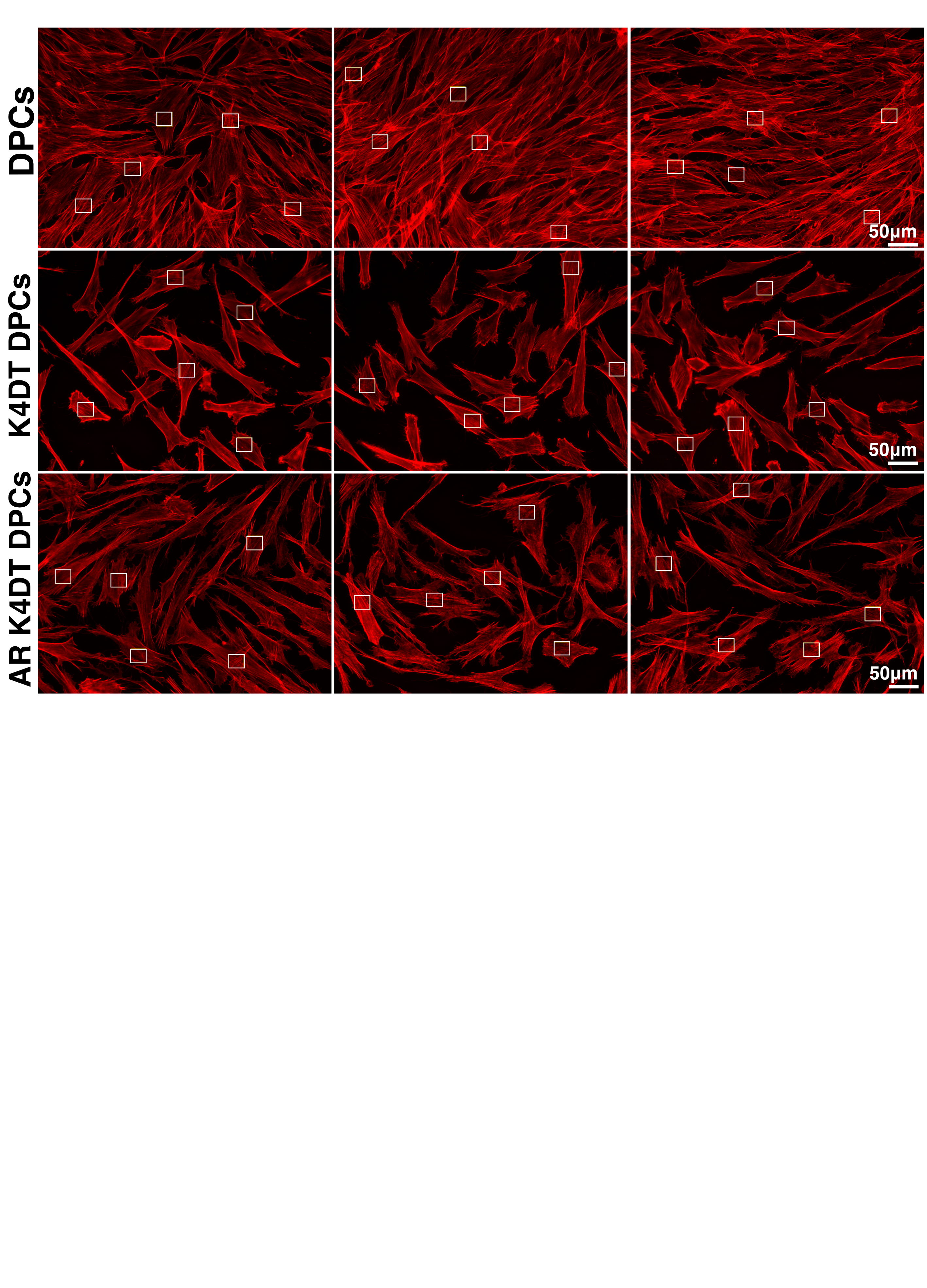

Supplement: FIGURE S5 — F-actin staining of measurement of fluorescence intensity of the 15 area. The area of the measurement of fluorescence intensity in wild type DPCs, K4DT DPCs, AR expressing DPCs were shown with white rectangles. [file Image_5.TIFF]

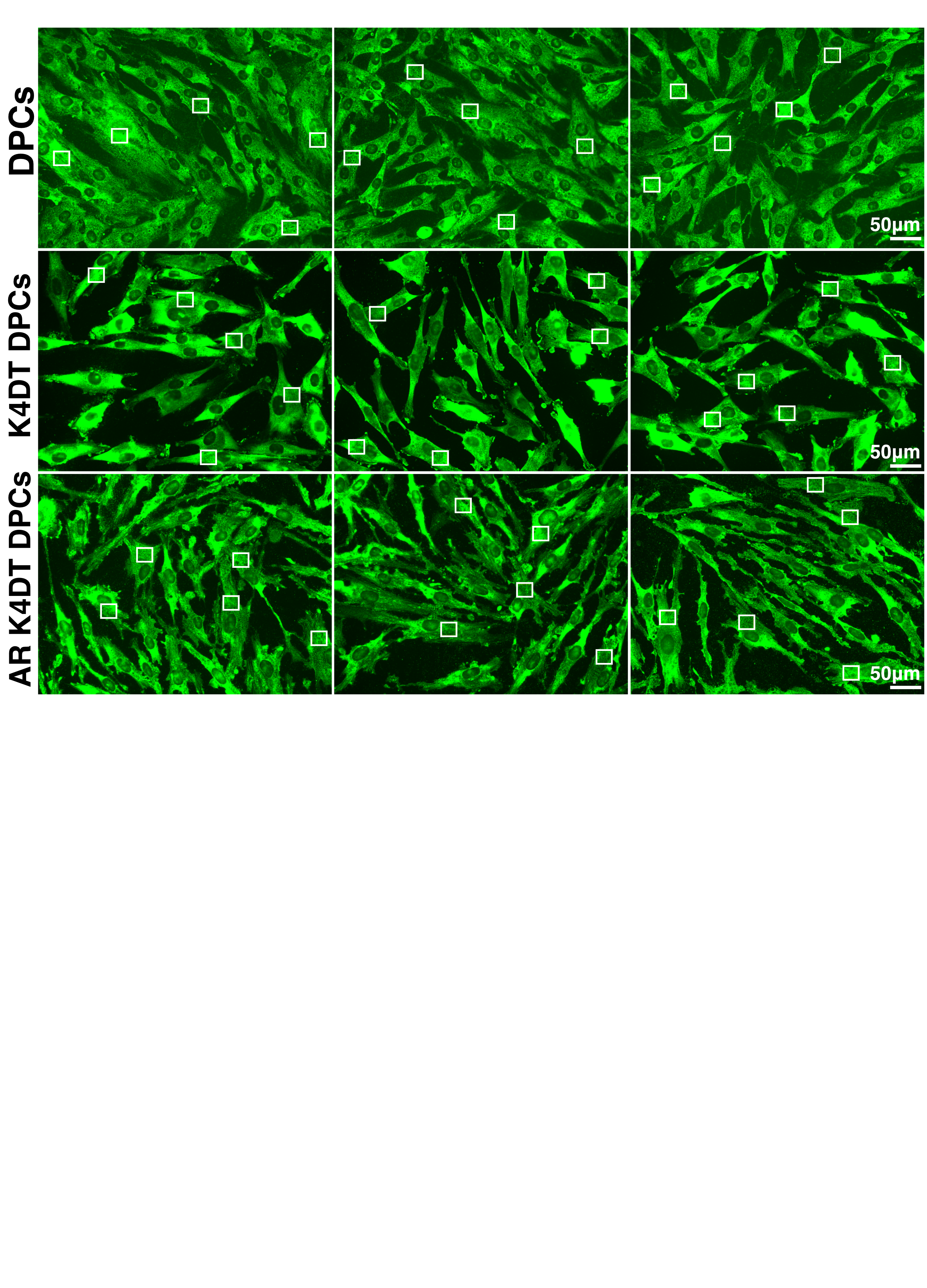

Supplement: FIGURE S6 — Immunostaining of α-smooth muscle actin (SMA) of wild type, K4DT, and AR expressing K4DT DPCs. The area of the measurement of fluorescence intensity in wild type DPCs, K4DT DPCs, AR expressing DPCs were shown with white rectangles. [file Image_6.TIFF]

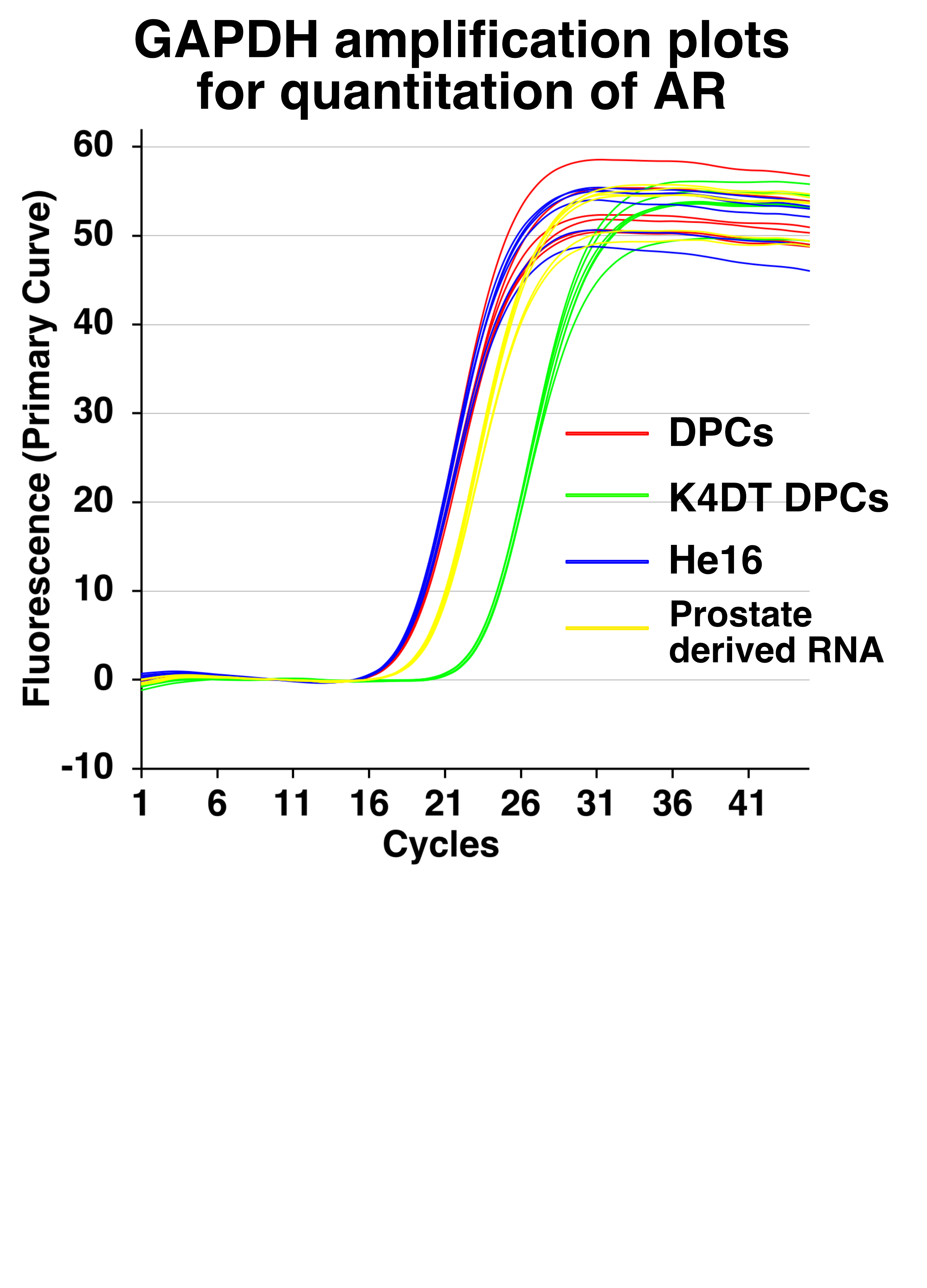

Supplement: FIGURE S7 — Amplification plots of androgen receptor (AR) with real time PCR analysis. Expression AR in wild type DPCs, K4DT DPCs, HE16, human normal prostate derived RNA were evaluated. [file Image_7.TIFF]

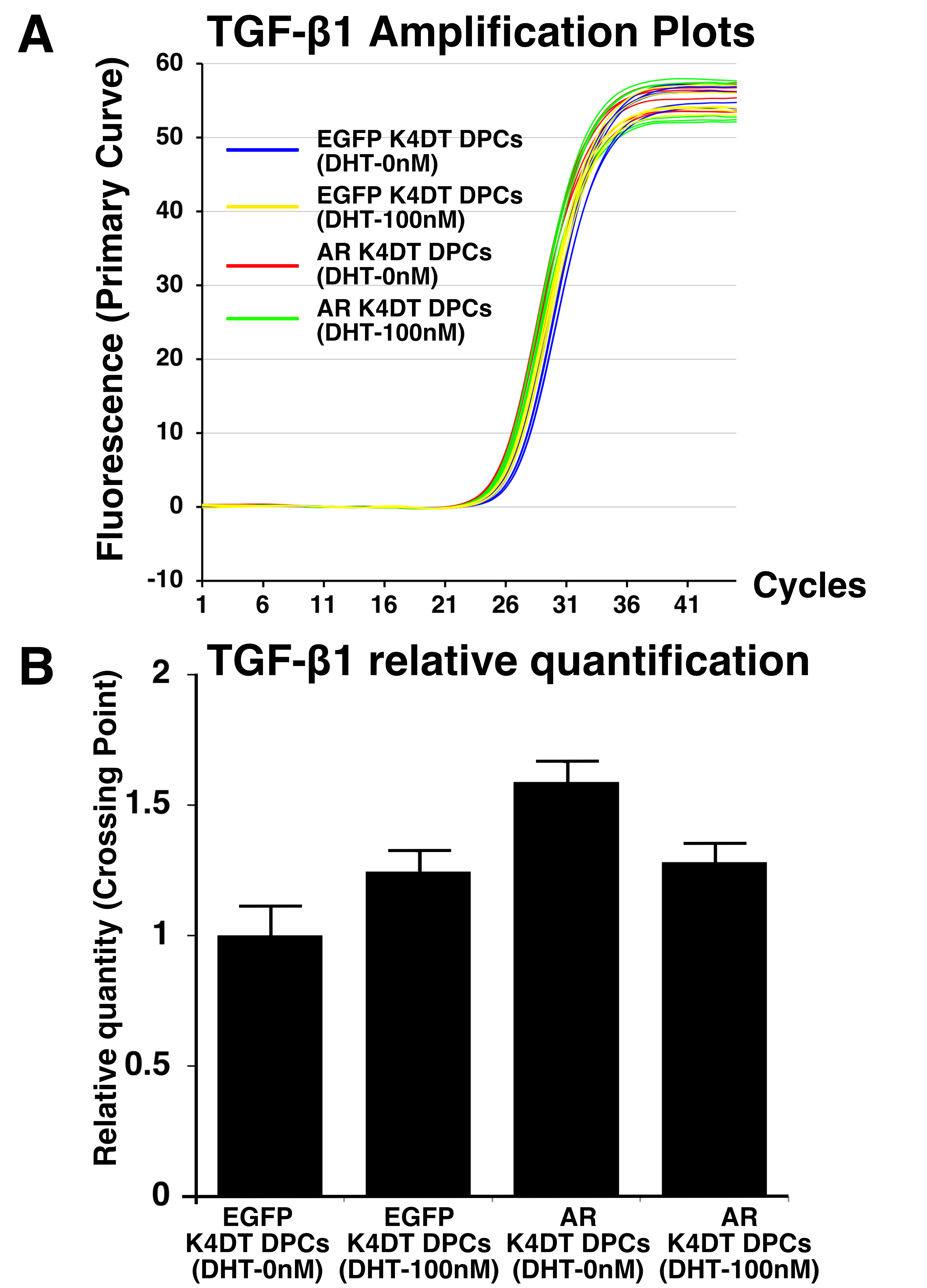

Supplement: FIGURE S8 — Amplification plots of TGFβ1 with real time PCR analysis. (A) Amplification plots of TGFβ1 in K4DT DPCs and AR expressing K4DT AR DPCs with and without dihydrotestosterone. (B) The quantitation of TGFβ1 expression with ΔΔ Ct method. [file Image_8.TIFF]

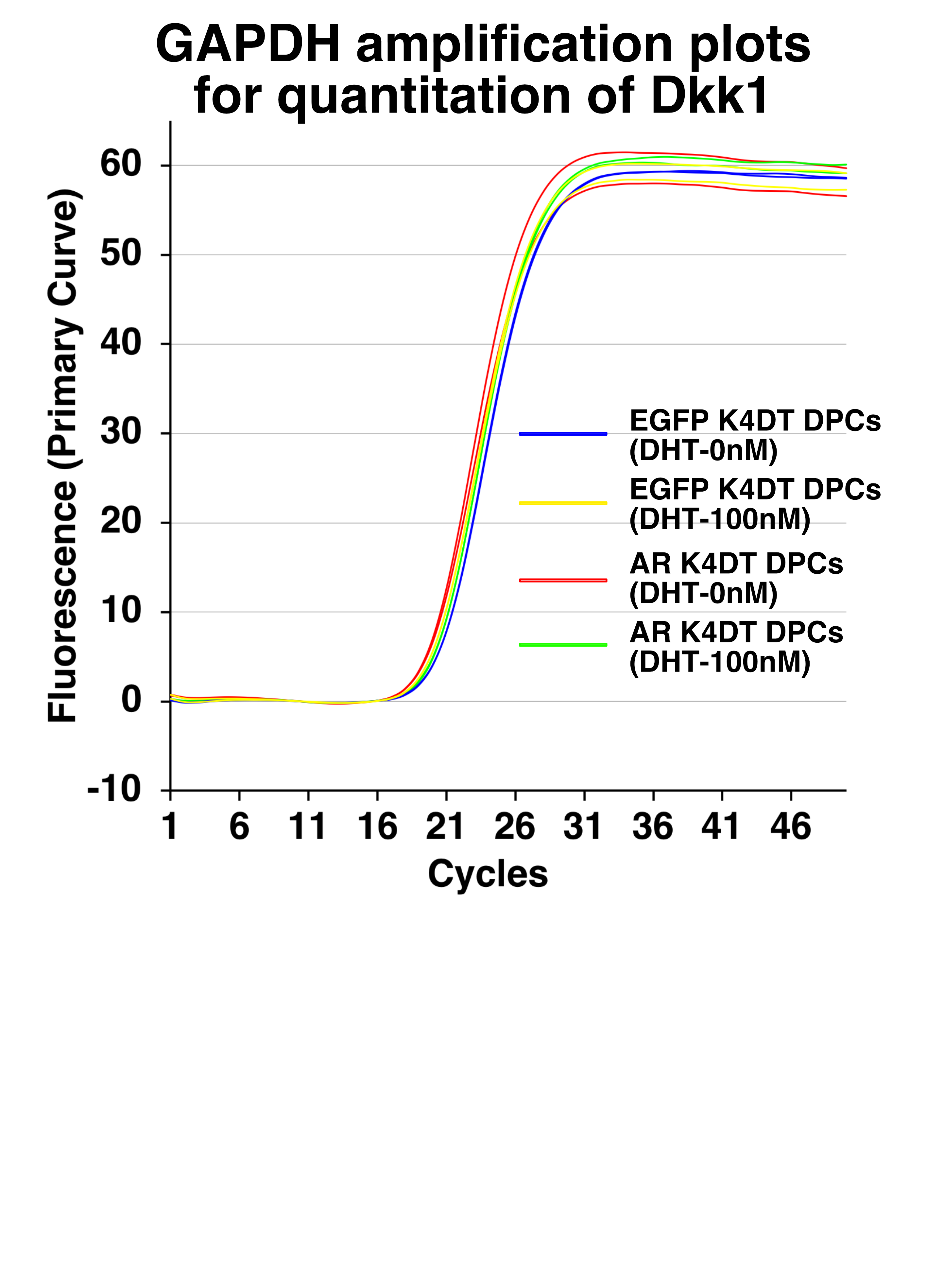

Supplement: FIGURE S9 — Amplification plots of glyceraldehyde-3-phosphate dehydrogenase (GAPDH) with real time PCR method for the quantitation of Dkk1. [file Image_9.TIFF]

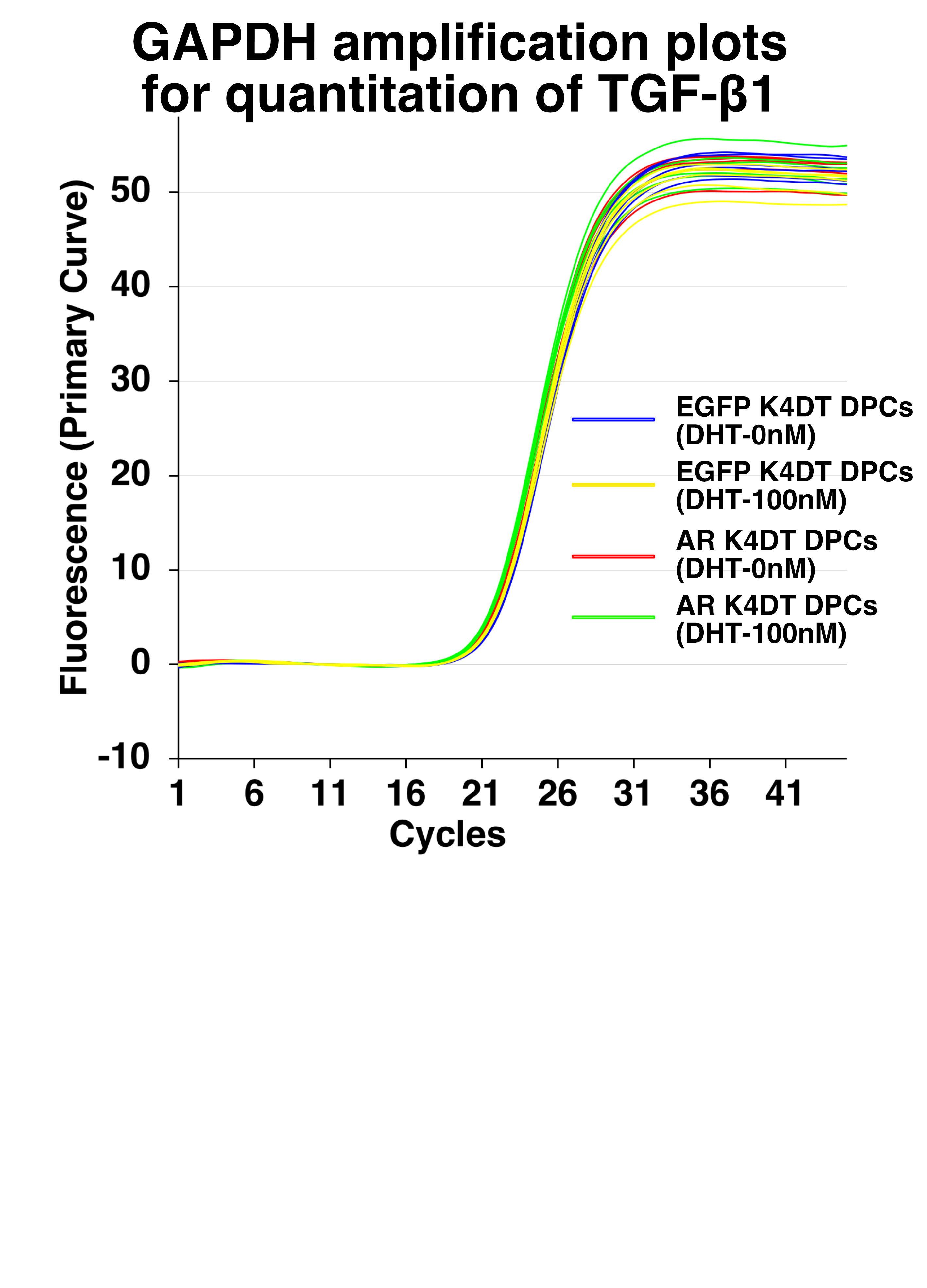

Supplement: FIGURE S10 — Amplification plots of glyceraldehyde-3-phosphate dehydrogenase (GAPDH) with real time PCR method for the quantitation of TGFβ1. [file Image_10.TIFF]
